# A comparative evaluation of deep learning models for the classification of encoded splice-junction sequences

**DOI:** 10.3389/frai.2026.1886470

**Published:** 2026-09-03

**Authors:** Yogesh Kumar, Inderpreet Kaur, Nandini Modi, Priya Bhardwaj, Jaeyoung Choi, Muhammad Fazal Ijaz

**Affiliations:** 1 Department of Computer Science and Engineering, School of Technology, Pandit Deendayal Energy University, Gandhinagar, India; 2 Department of Computer Applications, Chandigarh Group of Colleges, Mohali, India; 3 Department of Computer Science and Engineering, School of Computing, DIT University, Dehradun, India; 4 School of Computing, Gachon University, Seongnam-si, Republic of Korea; 5 School of Technology, Faculty of Business and Hospitality, Torrens University Australia, Melbourne, VIC, Australia

**Keywords:** benchmark dataset, bioinformatics, deep learning, model evaluation, splice-junction classification

## Abstract

**Introduction:**

Computational classifiers can be benchmarked against public encoded splice-junction datasets, but the performance estimate will depend on the input representation, preprocessing pipeline, model set-up and evaluation design.

**Methods:**

This study investigates the performance of 15 different Convolutional, Hybrid and Deep Learning architectures on a public encoded splice-junction dataset for 3-class classification. The results of the primary models were archived from one train/validation/held-out-test workflow and are presented as illustrative effects and not as statistically significant evidence of superiority.

**Results:**

The highest held-out test accuracy (96.87%) was obtained with the Custom CNN from among the configurations that were saved. The MLP Mixer was the most highly accurate model during training (99.90%), but the model did not perform as well on the validation set (93.33%) or held-out test set (93.73%), suggesting some in-sample fitting. Model-capacity information, parameter-to-sample ratios, learning curves, class-wise metrics, and a targeted class-weighting comparison are reported. Class-weighting was performed during the training phase only, but class-weighting did not yield highest performance for the tested Custom CNN configuration. of the chosen Custom CNN configuration for the observed dataset.

**Discussion:**

The conclusions are limited to the classification results that could be computed for the encoded benchmark dataset and the reported evaluation procedure. The results do not demonstrate a statistically significant superiority of the models in the sense of external generalization, clinical diagnostic validity, patient-based mutation detection, clinical utility or readiness for deployment. Leakage-free repeated evaluation, external datasets, stored sample predictions, statistically comparison of models’ outputs, and clinically validated patient-level data would be needed for more general conclusions.

## 1 Introduction

The genetic information is encoded in sequences of deoxyribonucleic acid (DNA) containing both exons and introns. The boundaries of exons and introns have been a longstanding challenge in computational genomics. The models are evaluated in the research stage on a public splice-junction dataset and no patient-level, diagnosis-specific or treatment-specific inference is made. Incorrect identification of splice junctions can lead to misfolded proteins associated with genetic disorders, including spinal muscular atrophy, cystic fibrosis, and some familial forms of cancer (Aarif et al., 2025; Bianchi, 2024). Although Sanger sequencing represented a major advancement in genetic analysis, it was slow and limited in scalability (Bianconi et al., 2023; Kumar et al., 2024; Satam et al., 2023), and therefore required replacement by newer and more advanced technologies that could analyze samples faster and at a more manageable scale, such as Next-Generation Sequencing (NGS) and Single-Molecule Real-Time (SMRT) sequencing. However, the complexity and high dimensionality of genomic data still present significant obstacles to correct interpretation, despite the scientific progress that has been made (Zaidi et al., 2023). Convolutional neural networks (CNNs) and long short-term memory (LSTM) networks have been used for computational investigation of genomic sequence patterns, such as analysis of cryptic and alternative splicing (Nakagiri et al., 2025; Fernandez-Castillo et al., 2022). The performance, however, is reported based on the dataset, the sequence representation, the validation procedure and the prediction task.

The rest of this paper is structured as follows. In Section 2, related studies of computational classification of splice-junctions are discussed. In section 3, the dataset, feature representation, data pre-processing and experimental design are described. In Section 4, the evaluated model architectures and their fixed, code-verifiable training configurations are presented. The experimental results presented in Section 5 refer to training, validation and held-out test performance. The results are compared with previous studies and limitations of the study are discussed in Section 6. Last, the conclusions and future research directions are summarized in Section 7.

### 1.1 Need for the study

The sequence classification of splice junctions is still a difficult problem, as the success rate is dependent on the input representation, the composition of the dataset, the preprocessing, the capacity of the model, and the validation procedure (Zabardast et al., 2023). The present work compares a variety of deep-learning architectures on the same encoded dataset, in terms of their observed training, validation and held-out test behaviour. It is not a clinical research and does not prove the detection of mutations or its clinical value for a particular disease. The following are the contributions of the study:

I The study compares a Custom CNN with CNN-based hybrid models, dense networks, TabNet, Wide & Deep, MLP Mixer, LSTM and an entity-embedding model in a task of sequence classification on the same encoded sequence.
II Standard preprocessing and regularization modules, such as early stopping, batch normalization (when applicable), and feature scaling, are used in the implementation. These components are defined as implementation decisions and the current archived record does not allow for a determination of a causal relationship between any one component and improved performance.
III Final training, validation and held-out test accuracy/loss and class-wise precision, recall and F1-scores are reported for 15 model configurations. The highest observed held-out test accuracy in the archived single-split evaluation is 96.87% for the Custom CNN. The MLP Mixer was trained with an accuracy of 99.90% and held out for testing with an accuracy of 93.73%.
IV The reported results are descriptive outcomes of just one split of a public dataset. These descriptive data should not be taken as evidence of statistical superiority of these models over others or over previous studies. They also lack evidence of disease specific mutation detection, clinical validity, clinical utility and deployment readiness.

### 1.2 Scope statement

The aim of this study is to compare, computationally, deep-learning, hybrid and conventional machine-learning configurations with a public encoded splice-junction benchmark dataset for a three-class classification problem. The reported test results for training, validation, held out are specific to the dataset, representation of the data, preprocessing procedure, fixed model configurations, and documented trial procedure. The primary model comparison analysis was performed with an archived single-split analysis and thus cannot establish model superiority, robustness across data sets, or generalization to an independent external data set. Additionally, there is no clinical information or clinically validated variant annotations at the patient level. Thus, the clinical diagnostic validity, patient-level mutation detection, clinical utility and deployment readiness are not evaluated in this study.

## 2 Related works

Previous computational research has used machine learning and deep learning techniques for classification of splice junctions or DNA sequences. Caution should be used when interpreting reported performance values for the following reasons: datasets, encodings, splits, label definitions, and validation designs are all different across studies. The purpose of the present review of the literature is to give methodological context to the literature, not to create a formal head-to-head comparison or basis for clinical claims (Baareh et al., 2021; Syahrani, 2019). This section examines deep learning methodologies for DNA sequence categorization and their utilization in genomic research, as shown in Table 1. The 94.8% accuracy attained by conventional machine-learning classifiers on the DNA Splice-Junction dataset is challenging to explain. While it required time-consuming parameter tweaks, the Primate Splice-Junction dataset showed a 3–5% improvement in performance when ensemble learning approaches were used (Syahrani, 2019), Random Forest and similar ensemble models execute prediction combination using majority voting which can be expressed mathematically as:

$$\hat{y}=\text{argma}x_{c\in C}\sum_{m=1}^{M}1[h_{m}\;(x)=c]$$ (1)

**Table 1:** Comparative analysis of DNA sequencing prediction.

Ref. no.	Dataset used	Techniques used	Results	Limitations
Baareh et al. (2021)	DNA splice-junction dataset	Machine learning classification	Accuracy: 94.8%, Precision: 93.5%, Recall: 95.2%, AUC: 96.7%	Model interpretability issues
Syahrani (2019)	Primate splice-junction dataset	Ensemble learning	Accuracy improvement of 3–5% over baseline models	Requires parameter tuning
Gautam et al. (2025)	Splice-junction gene dataset	CNN	Accuracy: 95.00%, Precision: 94.33%, Recall: 95.00%, AUC: 98.67%	Computationally expensive
Arputharaj et al. (2020)	Distributed DNA databases	Gene discretization model	Achieved a classification accuracy of 90%	Computational overhead
Imani et al. (2018)	Various DNA sequence datasets	Brain-inspired DNA processing	Higher than 90% accuracy	Complexity in model training
Fernandez-Castillo et al. (2022)	Human genome sequences	CNN	Achieved 96% precision	Requires extensive training
Nakagiri et al. (2025)	Human genome	Hybrid SVM-LSTM-RNN model	Precision of 96%	Requires computational optimization
Magnini et al. (2022)	Splice-junction gene dataset	AI-based gene Detection	Accuracy: 93.4%, Precision: 92.8%, Recall: 94.1%, AUC: 96.2%	Limited interpretability
Loka et al. (2019)	DNA sequence dataset	2D CNN on 1D data encoding	Achieved 96.5% accuracy	High computational cost
Anusuya and Gomathi (2021)	Splice-junction gene sequences dataset	Hybrid ML	Accuracy: 93.7%	Model complexity
Maigari et al. (2025)	GENE dataset (3,109 splice junction sequences)	Generative adversarial networks (GAN)	Accuracy: 96.8%, Precision: 97.5%, Recall: 96.2%, AUC: 98.4%	Requires large training data
Garouani et al. (2024)	Splice-junction gene sequences dataset	Model explainability in machine learning	Accuracy: 92.3%, Explainability Score: 87.5%, AUC: 95.1%	Accuracy vs. Interpretability Tradeoff
Imani et al. (2019)	Various DNA sequence datasets	Hyperdimensional computing	Achieved higher than 90% accuracy	Requires specialized hardware
Neighbor (2020)	Genome sequence classification dataset	Echo state networks (ESN)	Accuracy >90%	Model convergence challenges
Azmi and Berrado (2021)	DNA sequence data	Lasso-based pruning	Accuracy: 91.2%, Precision: 90.5%, Recall: 92.0%, AUC: 94.3%	Increased rule complexity
Zhang et al. (2023)	Various bioinformatics datasets	POP Choquet-like integrals	Accuracy: 95.2%, Precision: 93.8%, Recall: 94.5%	Computational complexity
Śmieja et al. (2019)	Primate splice-junction gene sequences	Mixture model clustering	Accuracy: 93.1%, Clustering Efficiency: 87.5%	Handling high-dimensional sparse data
Marchand et al. (2023)	DNA sequence classification	Sample re-attribution attack mitigation	Accuracy: 92.3%, Model Security Score: 89.8%	Privacy concerns in ML aggregation
Konovalenko (2019)	Splice-junction gene sequences	Heuristic-based ML algorithm	Accuracy: 94.7%, Precision: 92.9%, Recall: 93.6%	Model generalization limitations
Marton et al. (2023)	Bioinformatics DNA datasets	Gradient-based decision trees	Accuracy: 96.2%, F1-score: 94.8%, AUC-ROC: 95.5%	Computational complexity
Sharif et al. (2025)	DNA splice-junction dataset	Advanced machine learning framework	Accuracy: 96.8%, Precision: 95.9%, Recall: 96.4%, AUC: 97.6%	Limited validation across heterogeneous datasets
Hackl et al. (2026)	RNA-Seq splice junction reads	Deep learning and hybrid filtering	Accuracy: 95.6%, Precision: 94.9%, Recall: 95.1%, F1: 95.0%	Dependent on sequencing depth
Swaraja et al. (2025)	DNA splice-junction dataset	Transformer-based ensemble model	Accuracy: 97.2%, Precision: 96.8%, Recall: 97.0%, AUC: 98.1%	High computational cost
Jonathan et al. (2025)	Cancer fusion gene dataset	ML + hybrid sequencing approaches	Accuracy: 96.4%, Precision: 95.7%, Recall: 96.1%, AUC: 97.3%	Requires large annotated fusion datasets
Hong et al. (2026)	Multi-taxa splice-site dataset	Hybrid CNN-based deep learning	Accuracy: 96.9%, Precision: 96.2%, Recall: 96.7%, AUC: 97.9%	Reduced performance on low-coverage genomes
Linder et al. (2025)	Human genome sequences	Deep CNN regulatory model	AUPRC: 98.4%, AUC: 99.1%	Strong dependence on data volume

Some of the studies cited are used to provide methodological context only and the results of those studies are not interpreted as evidence of clinical validity nor as immediately comparable benchmarks for this dataset.

In this case, m is the number of classifiers, $h_{m}\;(x)$ is the class assigned by classifier m, and C is the set of classes, and 1[.] is the indicator function. Some previous studies mention relatively good classification metrics for CNN-based approaches. But it is difficult to make direct comparisons because of dataset differences, input encoding, different class definitions, partitioning of the datasets and the evaluation procedure. A CNN-based model processed the Splice-Junction Gene Dataset with 95% accuracy and 98.67% AUC, but its high computational requirements proved problematic, according to the authors in (Gautam et al., 2025). The convolution operation employed in CNN-based splice-junction models can be expressed as follows:

$$y_{i}^{\left(k\right)}=f(\sum_{m=0}^{k-1}\sum_{c=1}^{c_{\text{in}}}w_{m,cx_{i+m,c}}^{\left(k\right)}+b^{\left(k\right)})$$ (2)

In this case, i represents the position of the output, k represents the position of one of the filters of the output, k is the length of the kernel, $c_{\text{in}}$ is the number of channels in the input, and *f*(.) is the activation function. The same type of CNN analysis conducted on Human Genome Sequences reached 96% precision after intensive training (Fernandez-Castillo et al., 2022). A standard convolution block within deep splicer frameworks contains the sequence of convolution followed by ReLU activation and batch normalization and appears as:

h=BatchNorm[ReLU(Conv1D(x))] (3)

Some hybrid systems combine SVM-LSTM-RNN for sequence analysis, yet previous studies report precision values of approximately 96% (Nakagiri et al., 2025). The hidden state update in LSTM-based hybrid systems operates using this formula:

$$c_{t}=f_{t}⊙c_{t-1}+i_{t}⊙({\tilde{c}}_{t})\;h_{t}=o_{t}⊙\tanh(c_{t})$$ (4)

Here, f_t_,i_t_,and o_t_ are the forget, input, and output gates; c ~_t_ is the candidate cell state; and ⊙ denotes element – wise multiplication. The AI-based gene detection model trained on the Splice-Junction Gene Dataset produced 93.4% accuracy, but interpretability was limited (Magnini et al., 2022). A 2D CNN applied to 1D encoded DNA data achieved 96.5% accuracy, yet high computational cost remains a barrier (Loka et al., 2019). Hybrid ML models for Splice-Junction Gene Sequences achieved 93.7% accuracy, but their complexity makes implementation difficult (Anusuya and Gomathi, 2021). GAN-based generative models on the GENE dataset provided 96.8% accuracy with 97.5% precision yet required large-scale training data (Maigari et al., 2025). The adversarial training objective in GAN models can be formally written as a minimax game between the generator G and discriminator D:

$$\begin{array}{l} \begin{array}{c} \min\\ G \end{array}\begin{array}{c} \max\;\\ D \end{array}V(D,G)=E_{x∼\text{pdata}}[\log\;D(x)]\\ +E_{z∼\mathrm{pz}}[\log(1-D(G(z)))] \end{array}$$ (5)

Equations (1-5) summarize the principal mathematical formulations used to describe the ensemble, convolutional, recurrent, and adversarial learning operations discussed above.

However, lasso-based pruning (Liu et al., 2025) was able to attain an accuracy of 91.2%, yet the rules produced were complicated (Azmi and Berrado, 2021). Sample re-attribution defence strategies were also explored for better classification security and privacy (Marchand et al., 2023). ML-based on heuristics scored 94.7% accuracy, 92.9% precision and 93.6% recall, but had a problem with generalization (Konovalenko, 2019). Even though they have improved, issues such as computing demands, closed system designs, and suboptimal training remain a problem. The need for future studies is to give emphasis to leakage controlled preprocessing, repeated evaluation, independent external datasets, transparent reporting, and a correct model output interpretation validation. Another framework on a higher level of machine learning applied to splice-junction data of human DNA was proposed by (Sharif et al., 2025), with great accuracy but little validation on other heterogeneous datasets.

Based on the results of RNA-Seq, Hackl et al. (2026) used deep learning with hybrid filtering with high performance but was sensitive to the level of sequencing. Thereafter, Swaraja et al. (2025) presented an ensemble of detection of splice-junction in the DNA by means of transformer at the cost of high computing power. In cancer genomics, Jonathan et al. (2025) incorporated machine learning and hybrid sequencing to detect fusion gene, they needed large datasets that were annotated. In the meantime, Hong et al. (2026) comprised a hybrid CNN model using multi-taxa data and (Linder et al., 2025) used deep CNNs on human genomes with high predictive value but high data requirements. Prior studies are discussed only to provide methodological context. Because published studies differ in datasets, input encodings, class definitions, training procedures, data partitions, and evaluation metrics, their reported performance values are not treated as direct numerical benchmarks for the present evaluation.

## 3 Dataset description and preprocessing

**Figure 1:**
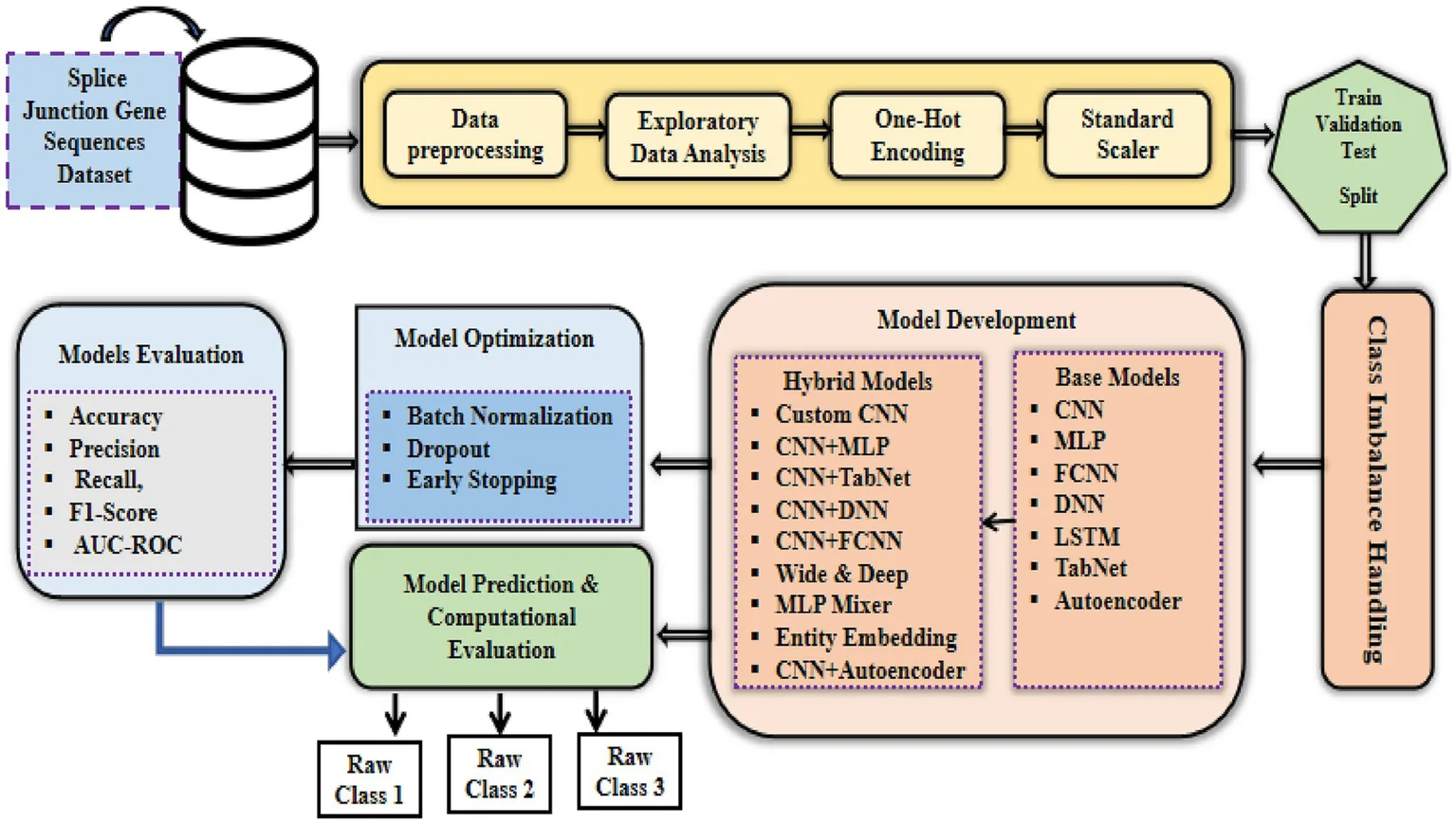
The research methodology for splice junction gene sequence classification.

The public encoded splice-junction dataset, the raw class distribution, the preprocessing workflow and experimental partitions used for the model development are described in this section. The analysis separated output codes from datasets, from clinically tested biological or diagnostic codes. The class-frequency imbalance is documented and an inverse-frequency class weighting is evaluated in the revised workflow with a corrected leakage-controlled training, validation and held-out test design. The entire experimental process from data preprocessing to model development, dealing with class imbalance, and computational assessments is illustrated in Figure 1.

The analysis of the dataset and descriptive statistics (EDA) are the initial stages of the workflow of the experiment, which are followed by feature encoding, normalization, and random train-test partitioning. These preprocessing streps aim to maintain the inherent biological features of splice-junction gene sequences and preprocess these data to be useful and trained in a model without bias.

### 3.1 Dataset provenance and analytical cohort

The data was retrieved from the UCI Machine Learning Repository. There were 3,190 instances, 60 categorical sequence-position variables, an instance identifier, and a class label in the source dataset. The analytical cohort consisted of 3,186 instances and 180 input variables (A0–A179), with the exception of the input variables that were preprocessed. 4 records were excluded because one or more positions in their sequences were ambiguous, non-canonical, nucleotide symbols (D, N, S, R). Nucleotide ambiguity is not missing data but these are the symbols used to represent ambiguity in the nucleotide before encoding. The original source labels were: ei, ie, and n. Each sample was subjected to the final analytical dataset of 3,186 samples. The number of samples in the provided analytical CSV file were 767 for class 1, 765 for class 2 and 1,654 for class 3. These labels are only used as a point of reference for the category of each dataset. They do not reflect patient diagnosis or clinically validated mutation types, disease mechanisms, or clinical utility. The feature representation and analytical scope of the reported experiments are summarized in Table 2.

**Table 2:** The feature representation and analytical scope of the reported experiments.

Component	Configuration used in the study	Analytical approach	Basis for interpretation	Relevance to reported results
Available encoded inputs	180 encoded columns, A0–A179	The complete encoded feature matrix was retained for model development	Columns were treated as computational input variables	The analysis used the full available encoded input space
Model input representation	Complete 180-feature matrix	Dense and tabular models used (180,); one-dimensional models used (180, 1)	Performance was assessed using the complete representation	Training, validation, and held-out test metrics reflected all 180 encoded features
Feature relevance assessment	Full-feature modelling approach	Model comparisons focused on architecture-level performance using the common encoded input representation	The study evaluated overall predictive performance rather than feature-level ranking	Results support comparison of model architectures under the same feature representation
Biological interpretation	Dataset-level encoded representation	Individual columns were not mapped to specific biological mechanisms within the present analysis	Biological interpretation requires externally validated feature annotation	Findings are limited to computational classification performance
Clinical interpretation	Dataset-specific benchmark labels	Outputs were evaluated as classification labels within the public dataset	Clinical interpretation requires patient-level evidence and external validation	Results should not be interpreted as clinical-marker or diagnostic evidence
Future extension	Feature-attribution and reduced-feature evaluation	Information gain, SHAP, permutation importance, and feature-subset comparisons may be incorporated in a future leakage-controlled workflow	Feature selection should be derived from training data and assessed independently	Future work can quantify the contribution of individual and reduced feature sets

Therefore, the conclusions about model performance presented in the reported results are based on the full 180 feature representation and cannot be taken as evidence of the predictive, biological or clinical significance of specific encoded columns.

### 3.2 Data preprocessing

**Figure 2:**
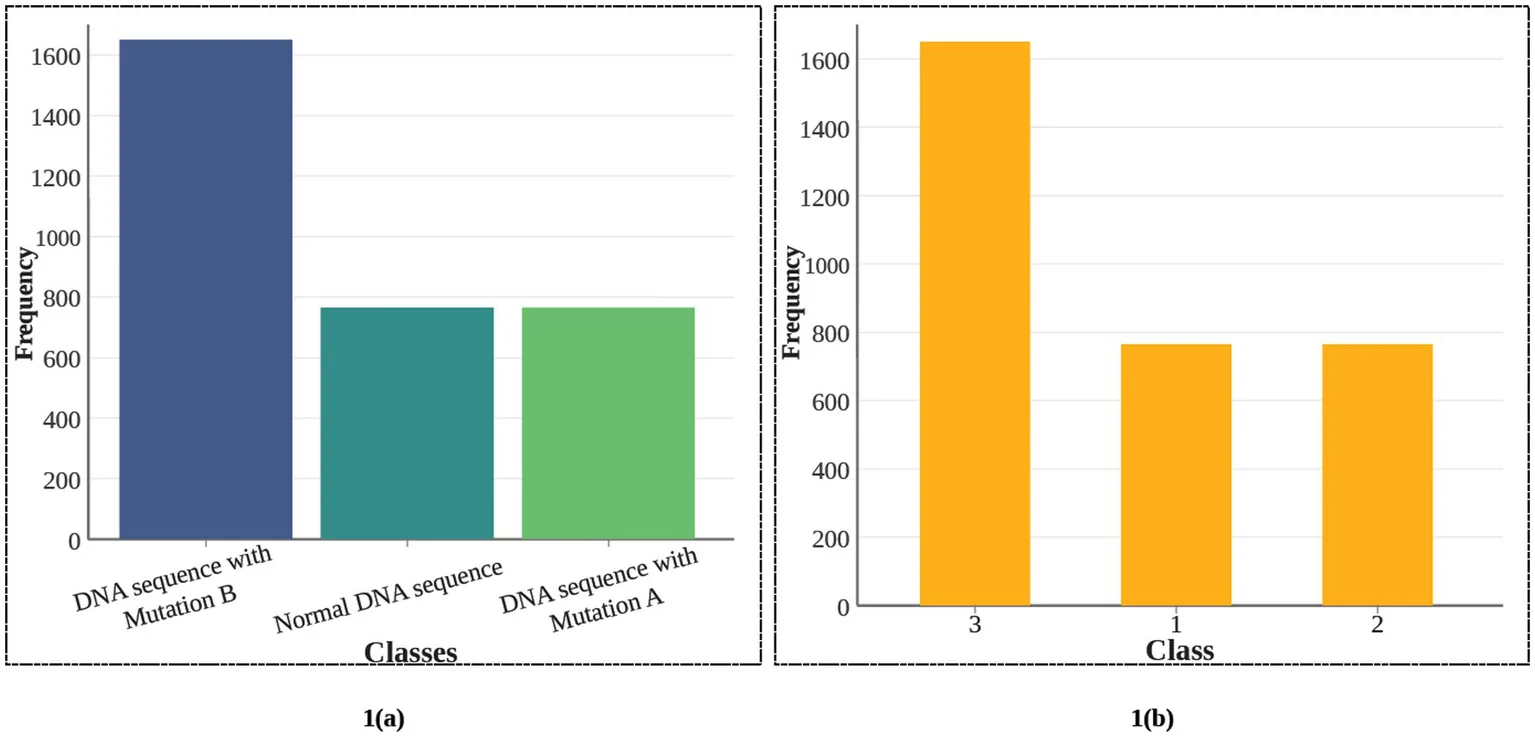
Distribution of raw class codes in the encoded splice-junction dataset.

The single-split results reported for the 15 model configurations were preprocessed with StandardScaler to standardise the encoded feature matrix before splitting the data into train/validation/test sets. So the test distribution held out, and this hidden distribution helped to scale the test parameters, which is a potential source of information leakage. The results presented, therefore, are taken simply as descriptive data. For the class-imbalance experiment discussed in Section 3.2.1 the data were first split into three partitions: training (64%), validation (16%) and held-out test (20%). Rather, the StandardScaler was only fitted on the training partition, and then fitted without refitting on the validation and held-out test partitions. Figure 2 depicts that there is a class-frequency imbalance in the analytical dataset, with encoded class 3 having more samples than encoded classes 1 and 2.

#### 3.2.1 Class-imbalance handling strategies

Three strategies to address class imbalance were systematically compared for the Custom CNN: (1) unweighted baseline, (2) inverse-frequency class weighting, and (3) random oversampling. Under the unweighted baseline strategy, all the training samples were equally important for the loss function. In the inverse-frequency class-weighting strategy, the minority classes were assigned higher weights according to their frequencies in the training partition, thus providing more weight to the errors of the minority classes. The random-oversampling method was used in which minority class samples were randomly duplicated in the training partition to balance the classes. This study did not employ any undersampling. Random oversampling was only used for the training dataset. The validation and held-out datasets were not oversampled and kept their class distribution. Oversampling was only done on the training set, thus avoiding information leakage and making sure the performance was measured on the test set following the original class distribution. The class weights were also determined based on the class frequencies in the training partition only. In this experiment, class-weighting was used in inverse fashion; the resulting class-weight dictionary was {0: 1.386395, 1: 1.386395, 2: 0.642092} with class 0 representing ei, class 1 representing ie and class 2 representing n. These weights have been embedded during training of the model with the loss function. Thus, error for classes 0 and 1 was more salient than that for class 2, the most common error. This class-weight dictionary was only applied to the inverse-frequency class-weighting experiment, and was not applied to the unweighted-baseline or random-oversampling experiment. This general architecture, optimizer, batch size, max number of epochs, stratified data partitions and random seed were all fixed between the three experiments, for consistency. Five stratified evaluations were repeated and the results are presented as mean ± std. in Table 3. The three assessed strategies, random oversampling, were found to have the highest mean balanced accuracy and macro-F1 score and thus were chosen as the class-imbalance handling strategy for the final Custom CNN configuration.

**Table 3:** Comparison of the unweighted baseline, inverse-frequency class weighting, and random-oversampling strategies.

Training strategy	Accuracy, mean ± SD	Balanced accuracy, mean ± SD	Macro-F1, mean ± SD	Weighted F1, mean ± SD
Unweighted baseline	0.9197 ± 0.0423	0.9387 ± 0.0285	0.9176 ± 0.0405	0.9200 ± 0.0428
Inverse-frequency class weighting	0.9129 ± 0.0473	0.9342 ± 0.0335	0.9112 ± 0.0459	0.9131 ± 0.0478
Random oversampling	0.9323 ± 0.0123	0.9458 ± 0.0088	0.9296 ± 0.0121	0.9329 ± 0.0119

Table 3 shows comparisons of the performance of three strategies to address class imbalance, an unweighted baseline, inverse-frequency class weighting, and random oversampling. Random oversampling was used for the training set only, while the class distribution remained the same for the test and validation sets. No under sampling was conducted. The class-weight dictionary reported in Section 3.2.1 was used only for the inverse-frequency class-weighting strategy.

#### 3.2.2 Feature-representation clarification and scope

All model results reported here used the full feature representation (with 180 input columns, A0 – A179). A set of A0–A10 was not included in the reported workflow as an experimental condition. In the reported workflow, no systematic feature-ranking or feature attribution analysis such as information gain, SHAP values, permutation importance or others was carried out. The biological relevance, clinical significance, and/or predictive importance of individual encoded columns was therefore not assigned by this analysis. Future work should focus on comparing the full 180 features with data-driven and predefined reduced feature subsets with a leakage controlled workflow. Only use training data for feature selection and then test the features on independent validation and held-out test data.

### 3.3 Experimental design and implemented training configuration

To compare, the targeted rerun of the Custom CNN was run with a corrected stratified split and training-only feature scaling. It applied corrected stratified split and training only feature scaling. This targeted class imbalance experiment was conducted in addition to the archived single split class-imbalance evaluation of the 15 model configurations.

#### 3.3.1 Data partitioning and training configuration

The archived results for the 15 deep learning architectures evaluated were obtained from a single train/validation/held-out test partition with a random seed of 42. Reported values of the performance are based on the fixed model configuration used in the attached source code. The results are given as descriptive results of the saved workflow and not as statistically validated performance results. The total feature set was standardized using the same workflow that was archived prior to splitting the data, and corrected train-only preprocessing was used for the additional class-imbalance experiment as described in Section 3.2. The settings that are reported in this study are only provided to enable the models assessed to be reproduced and are not interpreted as the result of a formal hyperparameter optimization procedure. There were 3,186 samples in the analytical dataset. The train_test_split(test_size = 0.20, random_state = 42) function used to split the data in the archived single-split workflow randomly selected 2,548 samples for the development set and 638 samples for the held-out test set. In the case of the Keras models, 20% (510 samples) of the development set was used for validation when the model was fitted; the development set included 2,038 samples. The 483, 474, and 1,081 (fitting), 131, 123, and 256 (validation), and 153, 168, and 317 (held-out test) classes for each encoded class were, respectively as shown in Table 4. This split is a random split, as the train_test_split call was made without an argument for stratification. There was no separate validation set provided to the fitting procedure for TabNet so that it was fitted on all 2,548 development samples. The results obtained for the single-split are archived and should not be confused with the extra experiment concerning class imbalance described in Section 3.2.1. The latter employed a separate stratified and leakage controlled method with the pre-processing and the treatment of imbalances being completely based on the training data.

**Table 4:** Exact sample counts in the archived single-split workflow.

Partition	Total	Class 1	Class 2	Class 3
Full analytical dataset	3,186	767	765	1,654
Keras fitting subset	2,038	483	474	1,081
Keras validation subset	510	131	123	256
Held-out test set	638	153	168	317
TabNet development set	2,548	614	597	1,337

## 4 Applied models for DNA sequencing prediction

The models tested in this study were computational architectures for the encoded input representation. The models are: convolutional, dense, recurrent, hybrid, Wide & Deep, TabNet, MLP Mixer and numerical-input classifier model and were compared on the same classification task related to a particular dataset. The architecture descriptions are in terms of input transformations, layer configurations, regularization components and output structures. They did not map each encoded input column to any biological function or clinical relevance or to any causal importance.

The feature matrix was reshaped to input format (180, 1) and then standardized before being fed into the LSTM model. It featured LSTM layers with 128 and 64 units, and a 128-unit dense layer and a 3-unit softmax output layer. A comparative baseline of an architecture-family, the recurrent model, was included. The experiment compared the reshaped encoded feature vector to a nucleotide-level sequential representation, but since the former was not a validated representation, there was no expectation that recurrent processing would give an advantage. The stacked 1D convolutional layers with the help of ReLU as an activation function and batch normalization was used in this model, supplemented by the reduced dimensionality of the output by max pooling and dropout (0.3) as a strategy to prevent the effects of overfitting. The last layer used a Softmax activation to specify the probability of the three classes as follows Raw class 1, Raw class 2, and raw class 3. Its learning was optimized with the Adam and categorical cross-entropy loss. The probability for class k was computed in the following way: $P(y=k|z)=\frac{\exp_{z_{k}}}{\sum_{j=1}^{3}\exp_{z_{j}}}$ , in which $z_{k}$ is the logit of class k produced by the last dense layer. Based on the concept of effective token interaction, the MLP Mixer model presented a non-convolutional model based on performing token-mixing (across positions within the DNA sequence) and channel-mixing (across features within the genome) using multilayer perceptrons. The MLP Mixer was added for comparison to a token- and channel-mixing architecture, which was used with the same encoded input representation as convolutional, dense, recurrent, and hybrid architectures. The reshaped input representation for shape (180,1) was fed to the implemented MLP Mixer. It applied the dense layer with 64 units using the ReLU activation function and then the dense layer with 128 units using the ReLU activation function for the channel mixing. The mixed representation was flattened and fed into a dense layer with 128 ReLU units and followed by three softmax units. The dropout rates used in the token- and channel-mixing blocks were 0.1, and the dropout rate before the output layer was 0.3. The archived implementation had no activation (GELU) or residual connections. In the archived implementation, token mixing was achieved with a 64-unit dense layer having ReLU activation and 0.1 dropout rate, followed by channel mixing with a 128-unit dense layer with ReLU activation and 0.1 dropout rate. The output of this model was flattened and passed through a 128-unit dense layer, and then classified into three classes using softmax. The Wide & Deep model was adopted in order to supplement models that only concentrate on either memorization of generalization. The Wide & Deep implementation used a learned deep representation in addition to the encoded feature vector. The concatenated representation was fed into a 128-unit dense layer and then through a 3-class softmax classification, while the deep path had dense layers with 128 and 64 units. The deep path contained three highly dense layers (128 → 64 → 3), each using ReLU as an activation function, and the output was calculated using Softmax. The overall prediction score was obtained by combining both a wide and a deep component: $\hat{y}=\text{softmax}(W_{\mathrm{wid}e^{x}}+W_{\text{deep}}(x)+b)$ where x is the input vector, a hidden representation h of the deep component and σ is the softmax function.

Further exploring the concepts of deep learning, the architecture of Fully Connected Neural Network (FCNN) was utilized as a model to learn nonlinearity feature transformation using dense layers of connections. Each of the neurons in one layer was connected with all the neurons in the other with the architecture parameters 128 → 64 → 32 → 3. A dropout and ReLU activation followed by softmax output made sure generalization as well as probabilistic classification. Forward propagation inside a hidden layer was performed according to the standard formula of a neuron activation: $a_{j}^{\left(l\right)}=\phi \;(\sum_{i}W_{\mathrm{ji}}^{\left(l\right)}a_{i}^{\left(l-j\right)}+b_{j}^{\left(l\right)}$ , where where ϕ is ReLU for the hidden layers. Lastly, the FCNN was extended into the Deep Neural Network (DNN) with even deeper learning capability that can learn more abstractive levels of gene signals. A deeper dense-network (DDN) baseline, which learned nonlinear transformations from the encoded input representation, was included as a baseline in the DNN. Similar to other models, it applied regularization based on dropout and optimization based on Adam. The softmax function was used to calculate the final class probabilities based upon the output in the last layer: $\hat{\mathrm{yk}}=\frac{e^{z_{k}}}{\sum_{i=1}^{3}e^{z_{i}w}}$ where $z=W^{\left(L\right)}h^{\left(L-1\right)}+b^{\left(L\right)}$ , where $z_{k}$ represent activation class of the k class, as a function of the weights $W^{\left(L\right)}$ and biases $\;b^{\left(L\right)}$ of the final layer. These architectures enabled a comparative evaluation of convolutional, dense, recurrent, mixer-based and hybrid architectures on the reported encoded input representation and training workflow.

### 4.1 Hybrid-architectures for genomic feature learning

Hybrid architectures are composed of different computational operations, namely convolutional feature extraction and dense / attention-based processing. For the present study, these architectures were added to the set of model configurations for comparison and were each supplied with an identical input representation in the form of encoding. Their inclusion does not indicate the superior performance, interpretability or clinical applicability. The CNN + MLP architecture is a hybrid model where convolutional layers are used to extract spatial or temporal features from unstructured inputs like images or sequential data. These features are then passed into a Multi-Layer Perceptron (MLP), which learns complex, nonlinear relationships and outputs final predictions. CNNs handle local feature extraction efficiently via shared weights and local receptive fields, while MLPs learn from the condensed features to make high-level decisions. This combination is widely used in image classification, biometric recognition, and scenarios where both spatial and non-spatial reasoning is needed.

The CNN branch is used to create a representation which is passed to the MLP branch, which is then trained with further non-linear transformations before classification. $\hat{y}=\mathrm{MLP}(\text{Flatten}(\mathrm{CNN}(X)))$ ,the estimated output is obtained by $\hat{y}$ using an input X passed through a CNN and the result flattened before being fed into an MLP. CNN + TabNet architecture works with multimodal data that contains unstructured representations (e.g., images) and structured tabular data. CNN serves to select deep feature representations in a given image data, whereas TabNet involves processes carried out on data that is tabular in nature with a group of sequential tabular modelling component. These two outputs are connected and inputted to the last decision layer with both types of data. TabNet was added as a tabular architecture, and the feature-level attribution results were not explored in the current study. Such a hybrid architecture is applied in such areas as medical diagnostics, remote sensing, or industrial monitoring. TabNet forms the prediction $\hat{y}$ with the combination of image-derived feature encoded through CNN in combination with raw tabular data, $\hat{y}=\text{TabNet}([\mathrm{CNN}(X_{\mathrm{img}}),X_{\mathrm{tab}}])$ .

Algorithm 1: **Hybrid CNN+MLP for splice-junction classification.**

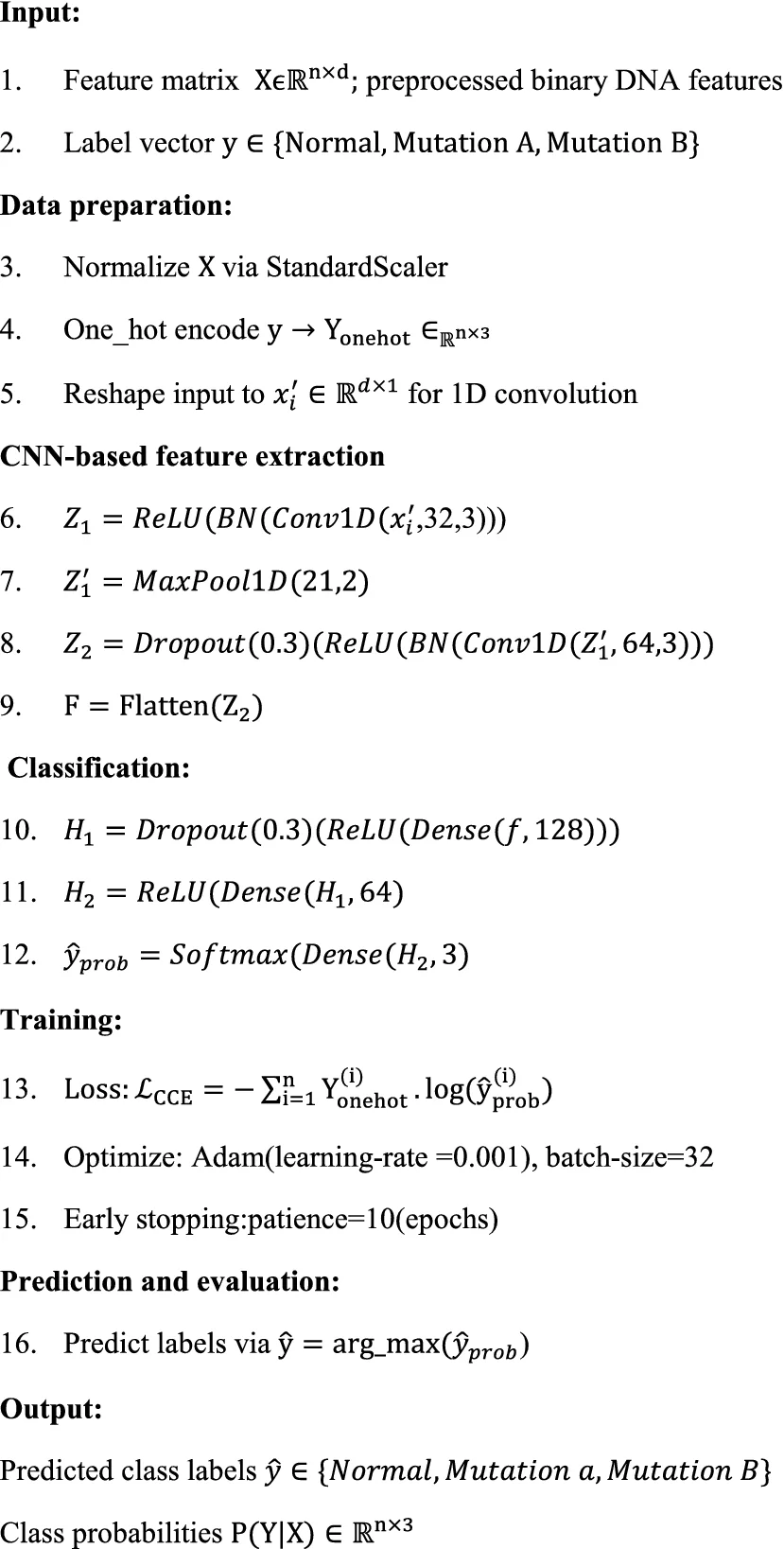

Algorithm 2: **Hybrid CNN+TabNet for splice-junction classification.**

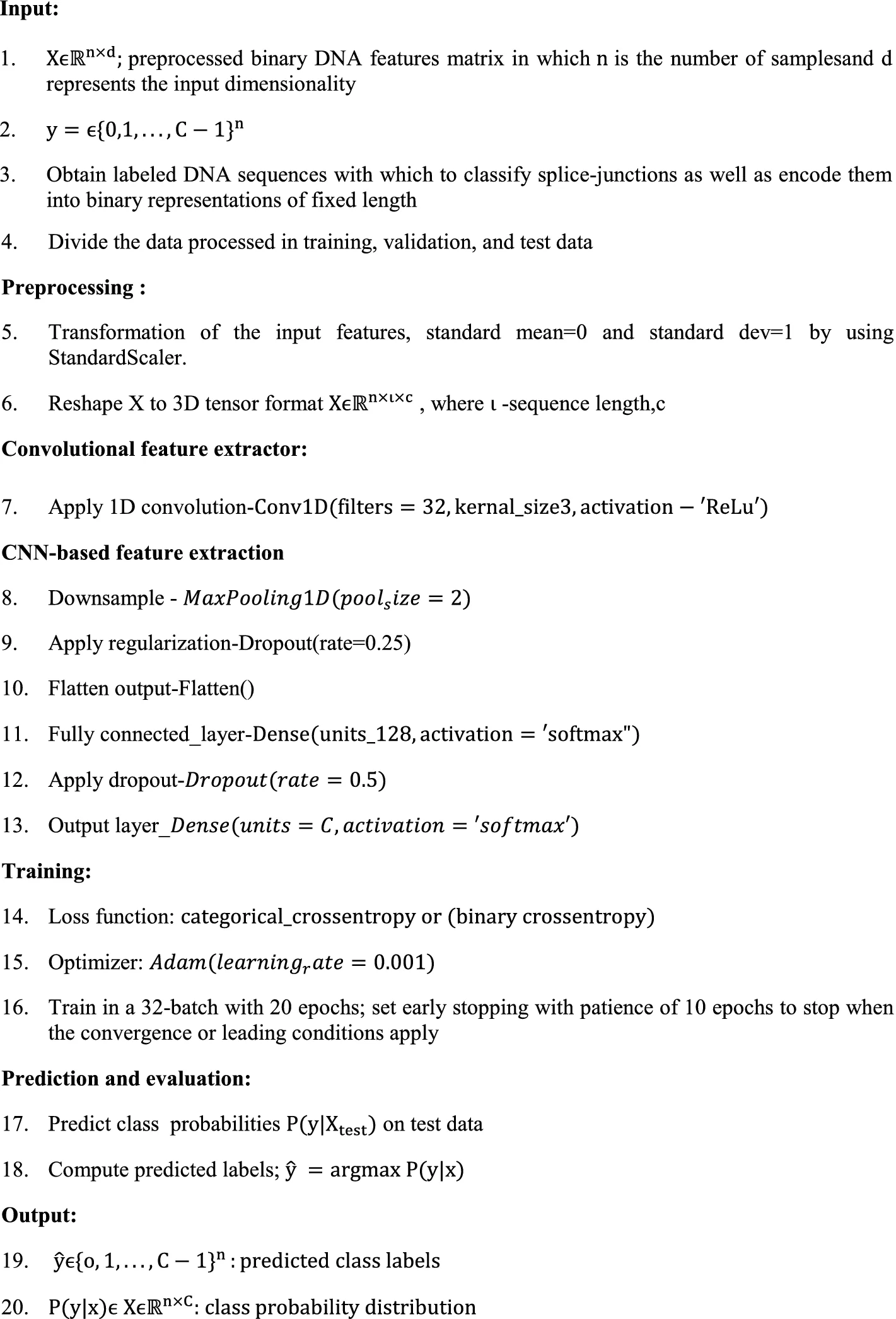

The Wide & Deep model is a combination of two different learning paradigms of a wide linear model and a deep neural network. The wide component does memorization through explicit-fitting feature-interaction via cross-product transformations. The deep component is generalized through acquisition of dense embedding and abstract features. The combination of them enables the model to reveal particular patterns as well as overall trends. The hybrid is particularly useful in recommendation systems, in advertising and in ranking tasks with some known interactions (wide) and others that must be identified (deep). Both the outputs are added and then sent through a sigmoid or softmax to give the final prediction, $\hat{y}=\sigma (W_{\mathrm{wid}e^{X}}^{T}+f_{\text{deep}}(x)),$ this equation represents Wide & Deep learning, in which linear terms $W_{\mathrm{wid}e^{X}}^{T}$ captures abstract patterns, summed and pass through σ ,to yield the final prediction $\hat{y}$ .

Algorithm 3: **Hybrid wide+deep for splice-junction classification.**

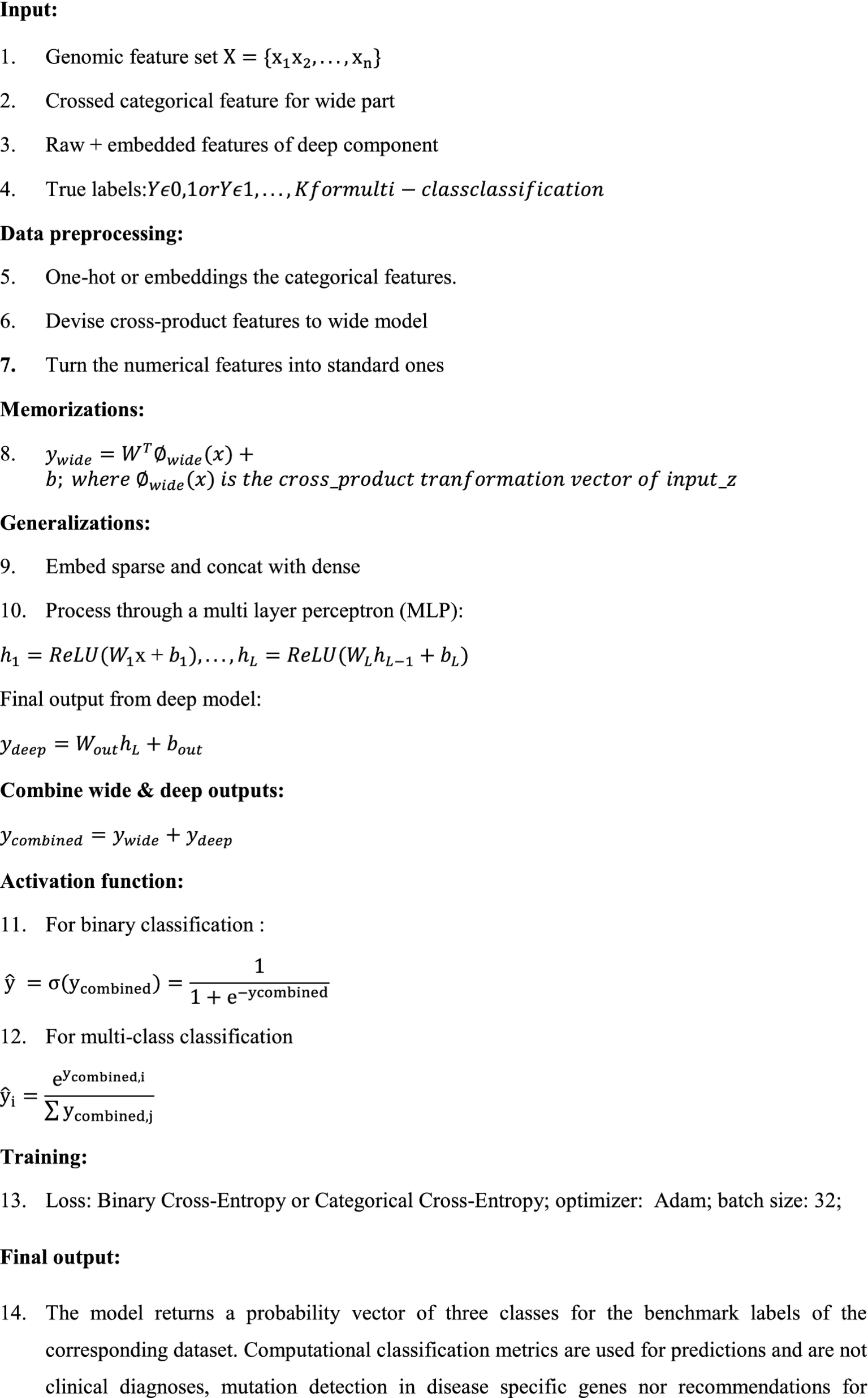

Overall, the hybrid models of CNN + MLP, CNN + TabNet, Deep + Wide mix the advantages of spatial feature extraction, non-linear modeling, and attention-based feature selection. CNN extracts patterns in genomic data that are localized, MLP increases this by providing complex relationships learning, and structured data processing and the Deep+Wide model allows the memorization of known feature interactions combining it with generalization using the deep representations. The hybrid configurations offer alternative ways of comparing the performance of the classification of the encoded splice-junction. They show only a performance of their results on the current benchmark dataset, and the clinical usefulness or biological significance of each of the variables encoded is not claimed.

### 4.2 Implemented model configurations and capacity analysis

The configurations of the evaluated models implemented and their corresponding model capacity are summarized in Table 5. The table lists only implementation parameters that can be derived directly from the provided source code, such as the input representation, main components of the architecture, number of trainable parameters, dropout parameters, batch normalization parameters and the number of samples to fit. This study does not assert any formal hyperparameter-search procedure, candidate search space nor optimization budget. Reported configurations are the fixed ones that have been implemented for generating the archived experimental results and are included here just for the sake of reproducibility.

**Table 5:** Implemented model configurations and model-capacity summary.

Model	Input shape	Key configuration from code	Trainable parameters	Non-trainable parameters	Fitting samples used	Parameters per fitting sample	Dropout/batch normalization
CNN	(180, 1)	Conv1D 32–64; Dense 128–3	3,59,107	0	2,038	176.21	Dropout: 0.2/0.2/0.3; no BN
TabNet	(180)	Default TabNet: (n_d = 8), (n_a = 8)	17,536	0*	2,548	6.88	Batch normalization; no dropout specified
FCNN	(180)	Dense: 256–128–64–3	87,683	0	2,038	43.02	Dropout: 0.3/0.3/0.3; no BN
MLP	(180)	Dense: 256–128–64–3	87,683	0	2,038	43.02	Dropout: 0.3/0.3/0.3; no BN
Autoencoder	(180)	Encoder: 128–64; classifier: 128–3	40,131†	0	2,038	19.69	Dropout: 0.2/0.2/0.2; no BN
LSTM	(180, 1)	LSTM: 128–64; Dense: 128–3	1,24,675	0	2,038	61.18	Dropout: 0.2/0.2/0.2; no BN
Custom CNN	(180, 1)	Conv1D: 32–64–128; Dense: 128–3	3,59,683	448	2,038	176.49	Dropout: 0.2/0.3/0.4/0.5; BN after each convolution
DNN	(180)	Dense: 512–256–128–3	2,57,283	0	2,038	126.24	Dropout: 0.3/0.3/0.3; no BN
CNN + MLP	(180, 1) + (180)	CNN 32–64 + MLP 128–64; fusion Dense 128–3	3,98,723	0	2,038	195.64	Dropout: 0.2/0.3 in CNN; 0.3 in MLP/fusion; no BN
CNN + FCNN	(180, 1) + (180)	CNN 32–64 + FCNN 256–128; fusion Dense 128–3	4,54,723	0	2,038	223.12	Dropout: 0.2/0.3 in CNN; 0.3 in FCNN/fusion; no BN
CNN + DNN	(180, 1) + (180)	CNN 32–64 + DNN 512–256–128; fusion Dense 128–3	6,32,387	0	2,038	310.3	Dropout: 0.2/0.3 in CNN; 0.3 in DNN/fusion; no BN
CNN + TabNet	(180, 1) + (3)	TabNet probability vector + CNN 32–64; fusion Dense 128–3	393,539‡	0*	2,548/2,038‡	6.88/184.50‡	CNN dropout: 0.2/0.3; fusion dropout: 0.2/0.3; TabNet uses BN
Wide & Deep	(180) + (180)	Raw-feature branch + deep path 128–64; fusion Dense 128–3	63,171	0	2,038	31	Dropout: 0.3/0.3/0.3; no BN
MLP mixer	(180, 1)	Token Dense 64; channel Dense 128; Dense 128–3	10,60,931	0	2,038	520.57	Dropout: 0.1/0.1/0.3; no BN
Entity embedding	(180)	Numerical Dense: 128–64–3	31,619	0	2,038	15.51	Dropout: 0.3/0.3; no BN

All reported model results were obtained with the full (encoded) feature representation, consisting of 180 input columns A0-A179.* TabNet parameter counts are reported using the implementation/framework parameter-counting convention; no separate non-trainable parameter count was recorded in the model summary.† The value 40,131 is the reported trainable-parameter count for the implemented autoencoder-based classification pipeline used for the reported result.

A total of 2,038 samples were used to fit parameters to the Keras-based models, with 510 samples from the 2,548-sample development set being assigned to the validation set. All 2,548 development samples were used to fit TabNet. CNN + TabNet is a CNN-fusion model fitted separately for CNN and TabNet, thus phase-specific sample counts and ratios are reported. As shown in Table 3, the unweighted baseline, inverse-frequency class-weighting, and random-oversampling strategies resulted in different classification performance levels. The unweighted baseline served as a baseline for comparing the effect of the two class-imbalance treatments. Both inverse-frequency class weighting (IFCW) and random oversampling (ROS) adjusted the contribution of each class to the loss function, but did not alter the number of samples used to train the model. Inverse-frequency class weighting (IFCW) and random oversampling (ROS) both weighted the contribution of each class to the loss function, but did not modify the number of samples used for training. The reported evaluation metrics (accuracy, precision, recall, F1-score, balanced accuracy, or AUC) were used to evaluate the comparative results.

#### 4.2.1 Custom CNN configuration and study contribution

The Custom CNN is not proposed as a new neural-network architecture. It is a convolutional architecture, which is a subset of the convolutional networks to be implemented, and it is part of a comparative study of set pre-designed deep learning architectures. Three Conv1D blocks (32, 64, 128 filters) are followed by batch normalization after every Conv1D block, max-pooling, progressive drop-out, and a 128 unit dense classification layer with a softmax output of three classes. The novelty of this study is that this configuration is compared to CNN, dense, hybrid, recurrent, tabular and mixer-based models on the same encoded splice-junction benchmark dataset. The findings should therefore be considered as observations on the specific configuration in the context of the present dataset and evaluation method, and not a proof of the existence of a new general-purpose CNN architecture.

### 4.3 Implemented training configuration

The architectures of deep learning models evaluated were implemented with fixed training configurations, determined during model development. The optimizer, the learning rate, the batch size, the number of epochs for training, the dropout configuration, batch normalization, the activation functions or the early stopping are directly taken from the corresponding source code and form part of the implementation settings reported in this study as shown in Table 6. The experimental results in Section 5 are reported using these settings. The following experiments were not performed or documented with any formal hyperparameter optimization method like Grid Search, Random Search, Bayesian Optimization, Optuna, or other automated search methods. The authors do not therefore claim that the configurations implemented were systematically optimized. Instead, the study should be seen as a comparative analysis of the fixed model configs, applied to the encoded splice-junction benchmarks dataset.

**Table 6:** Implemented training configuration used for the evaluated deep learning models.

Parameter	Implemented setting
Optimizer	Adam
Loss function	Categorical cross-entropy
Learning rate	0.001
Batch size	32
Maximum epochs	20
Early stopping	Patience = 10 (where implemented)
Activation function	ReLU (hidden layers), Softmax (output layer)
Batch normalization	Applied in architectures where implemented
Dropout	Architecture-specific (see Table 5)
Random seed	42
Input representation	Full 180 encoded features

## 5 Experimentation and evaluation

**Figure 3:**
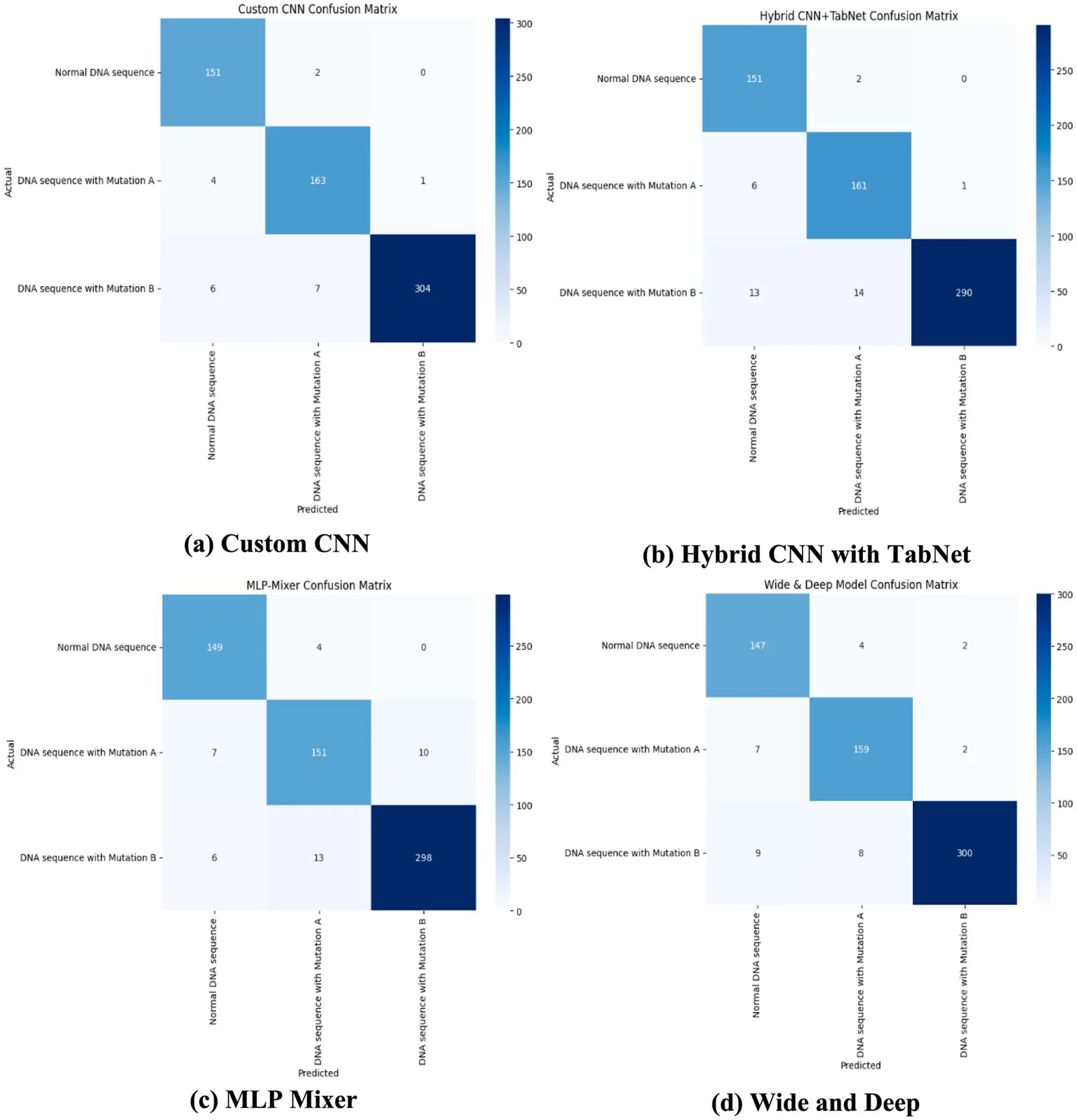
**(A–D)** Confusion matrix of classification models.

**Figure 4:**
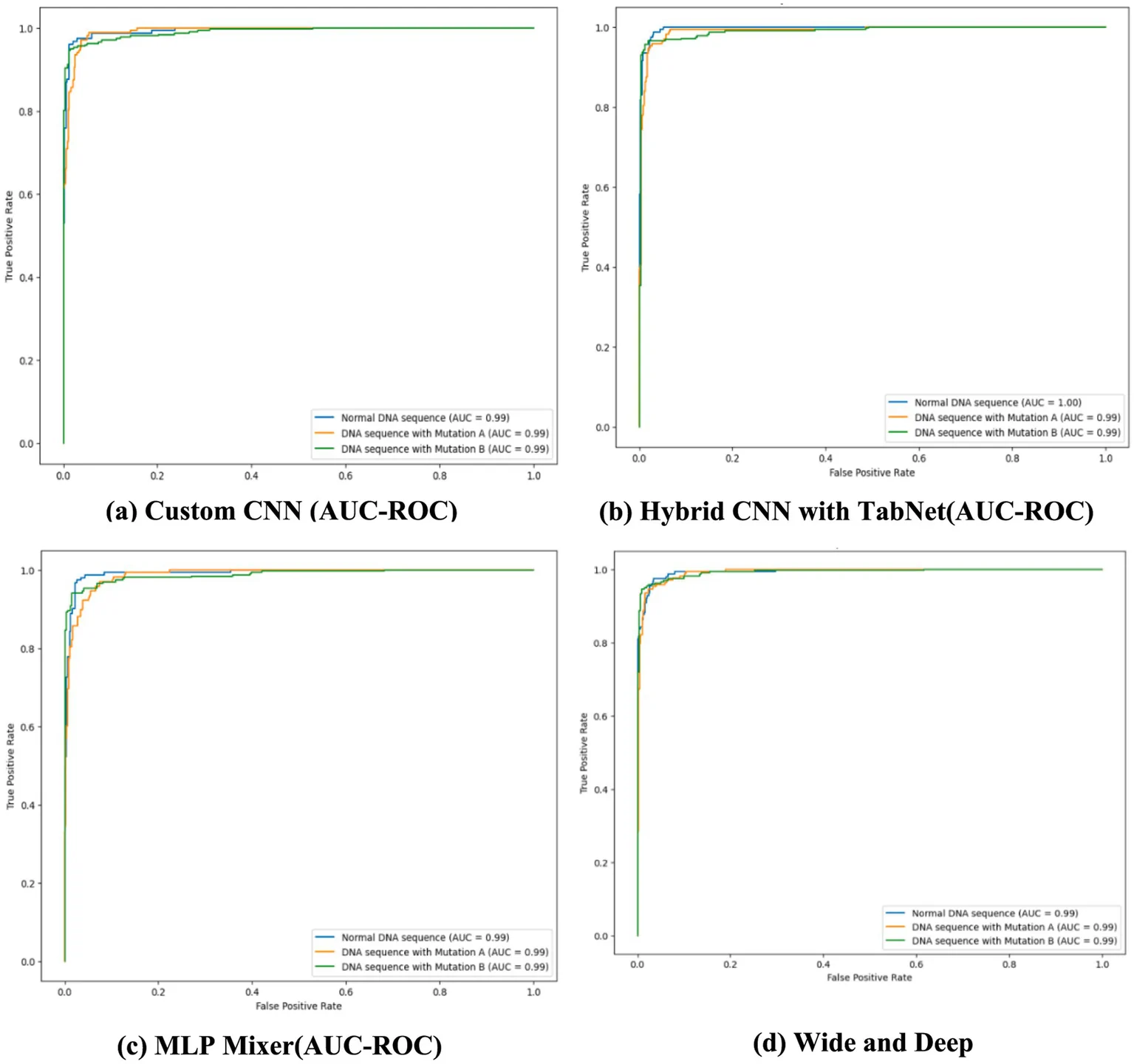
**(A–D)** AUC-ROC curves of classification models.

**Figure 5:**
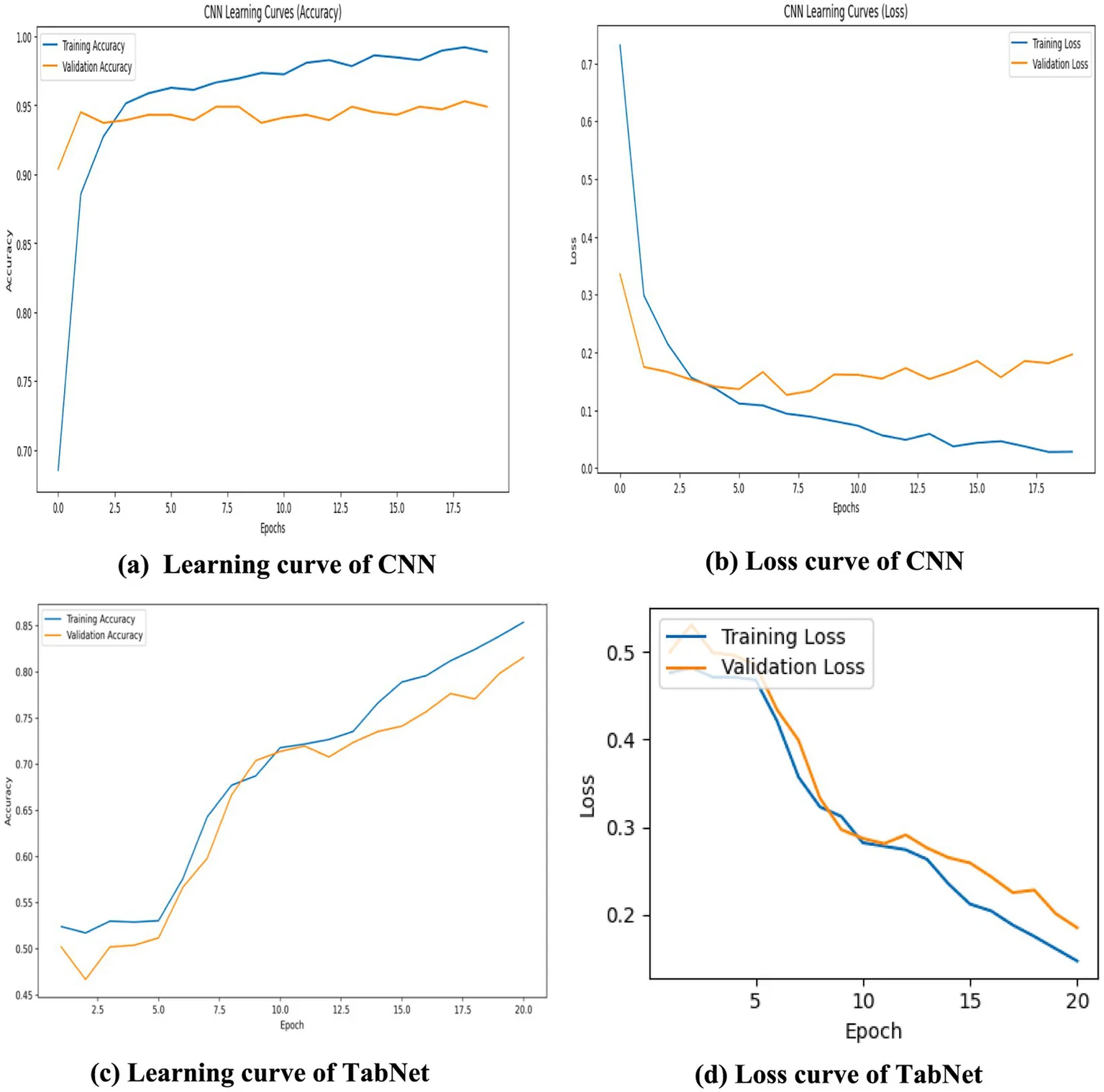
**(A–D)** Learning curves of CNN and TabNet models.

**Figure 6:**
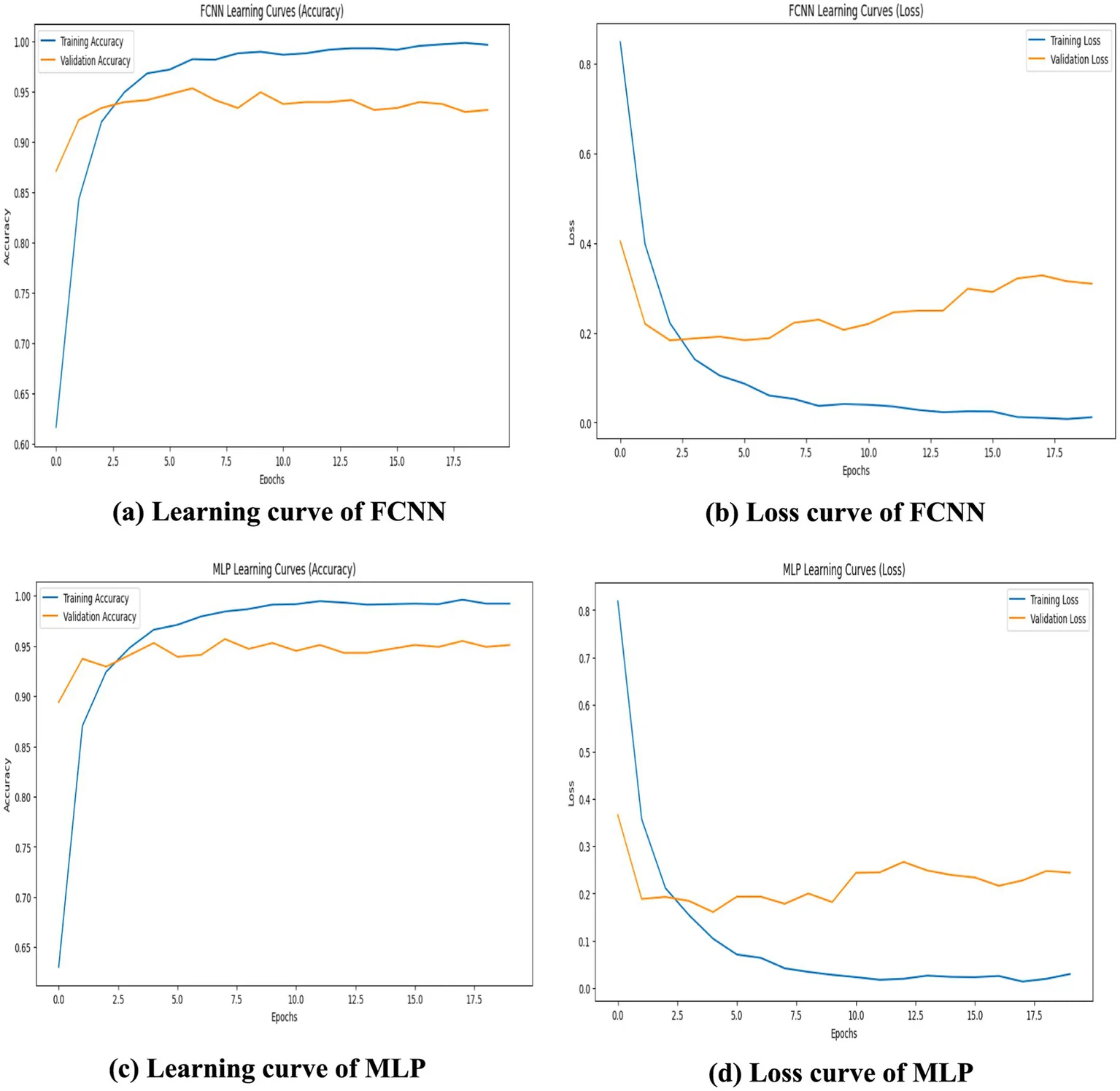
**(A–D)** Learning curves of FCNN and MLP models.

**Figure 7:**
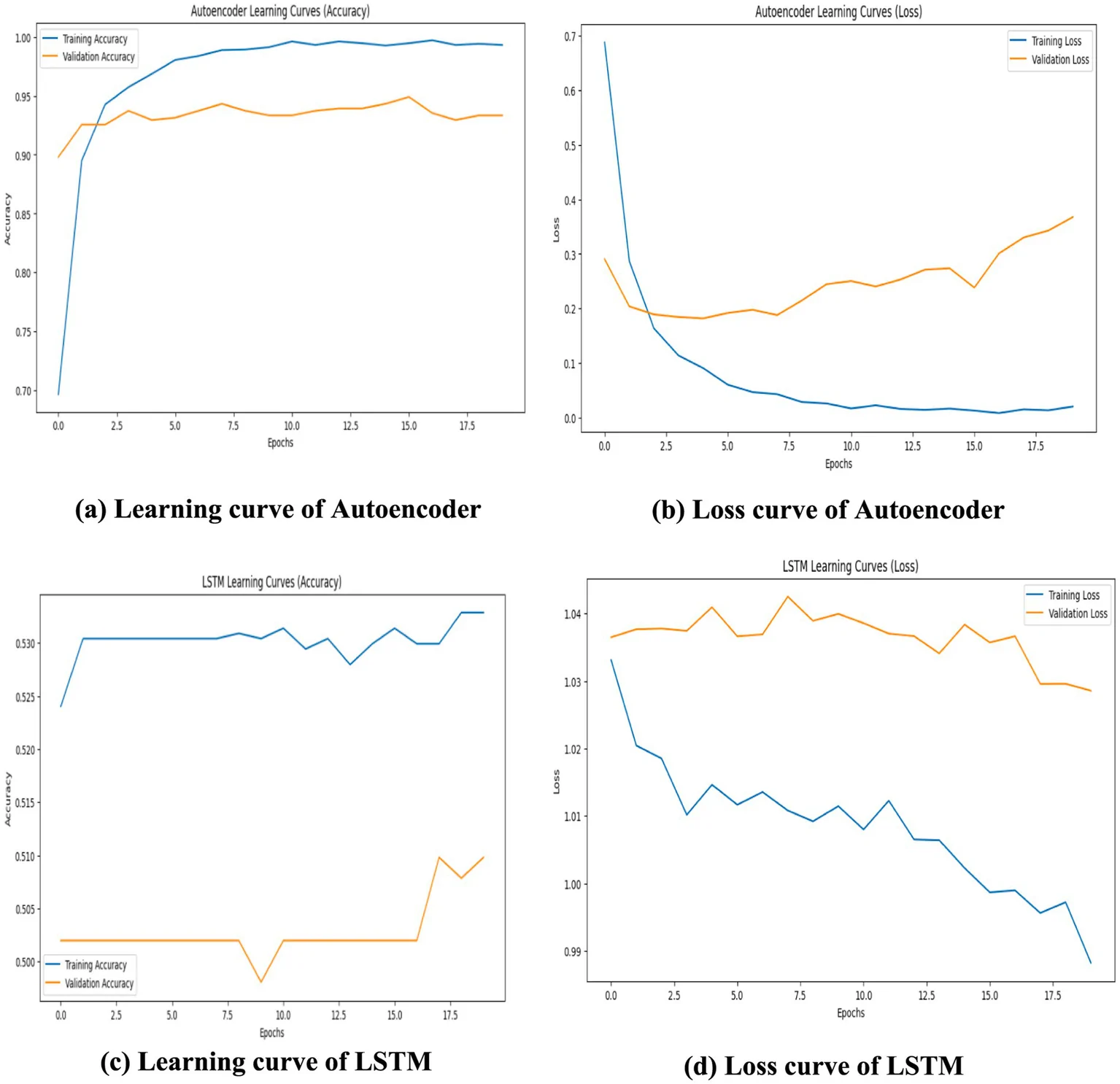
**(A–D)** Learning curves of Autoencoder and LSTM models.

**Figure 8:**
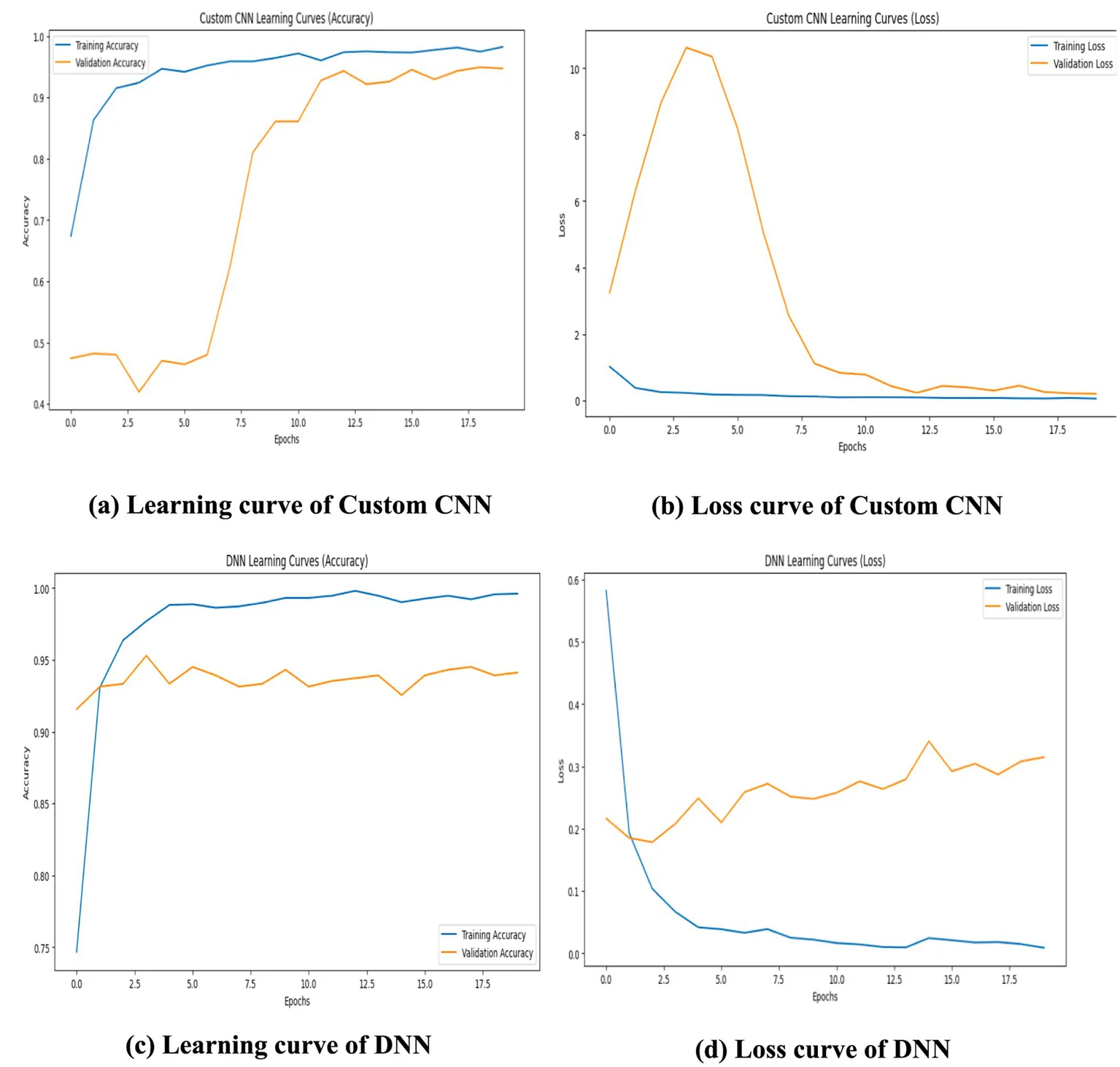
**(A–D)** Learning curves of Custom CNN and DNN models.

**Figure 9:**
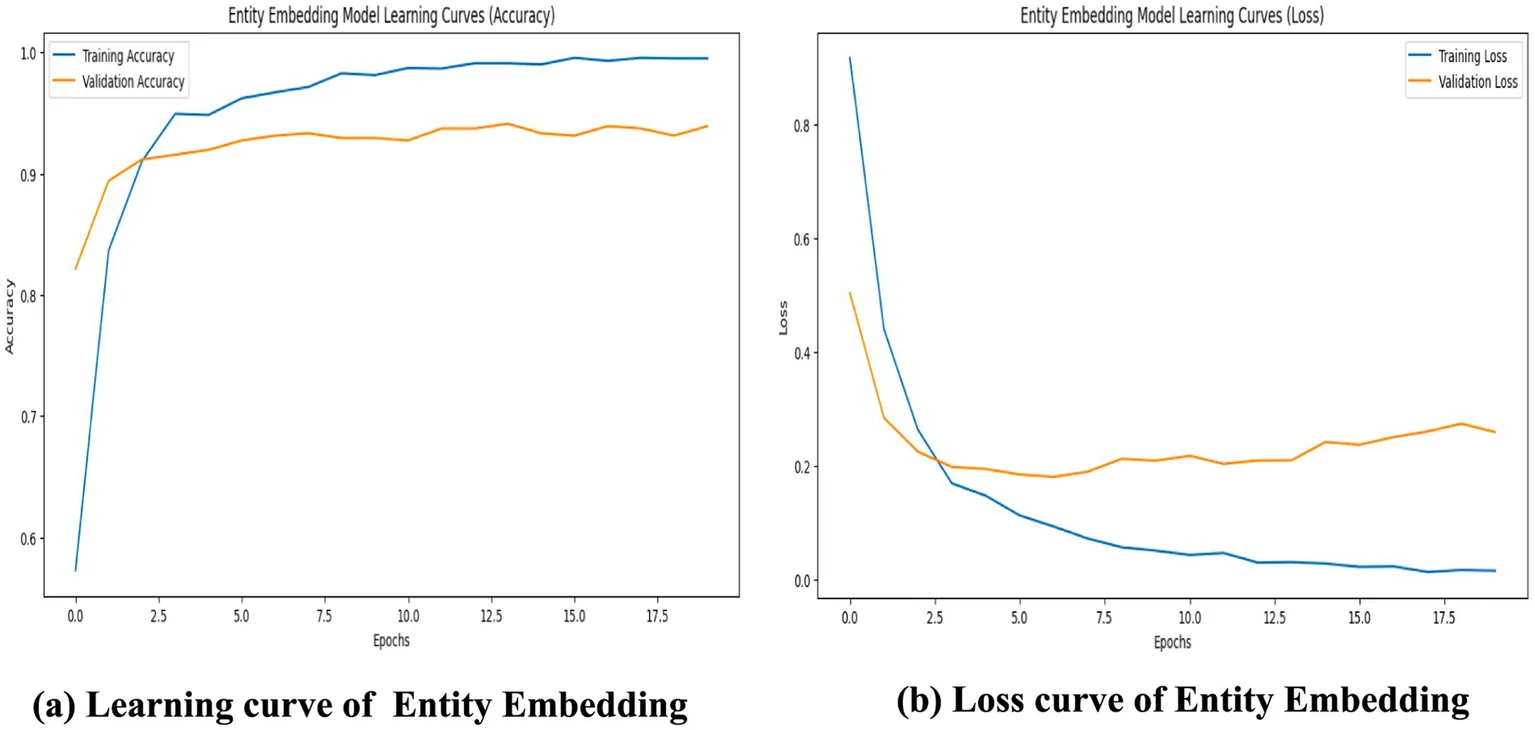
**(A–D)** Learning curves of the Entity Embedding model.

**Figure 10:**
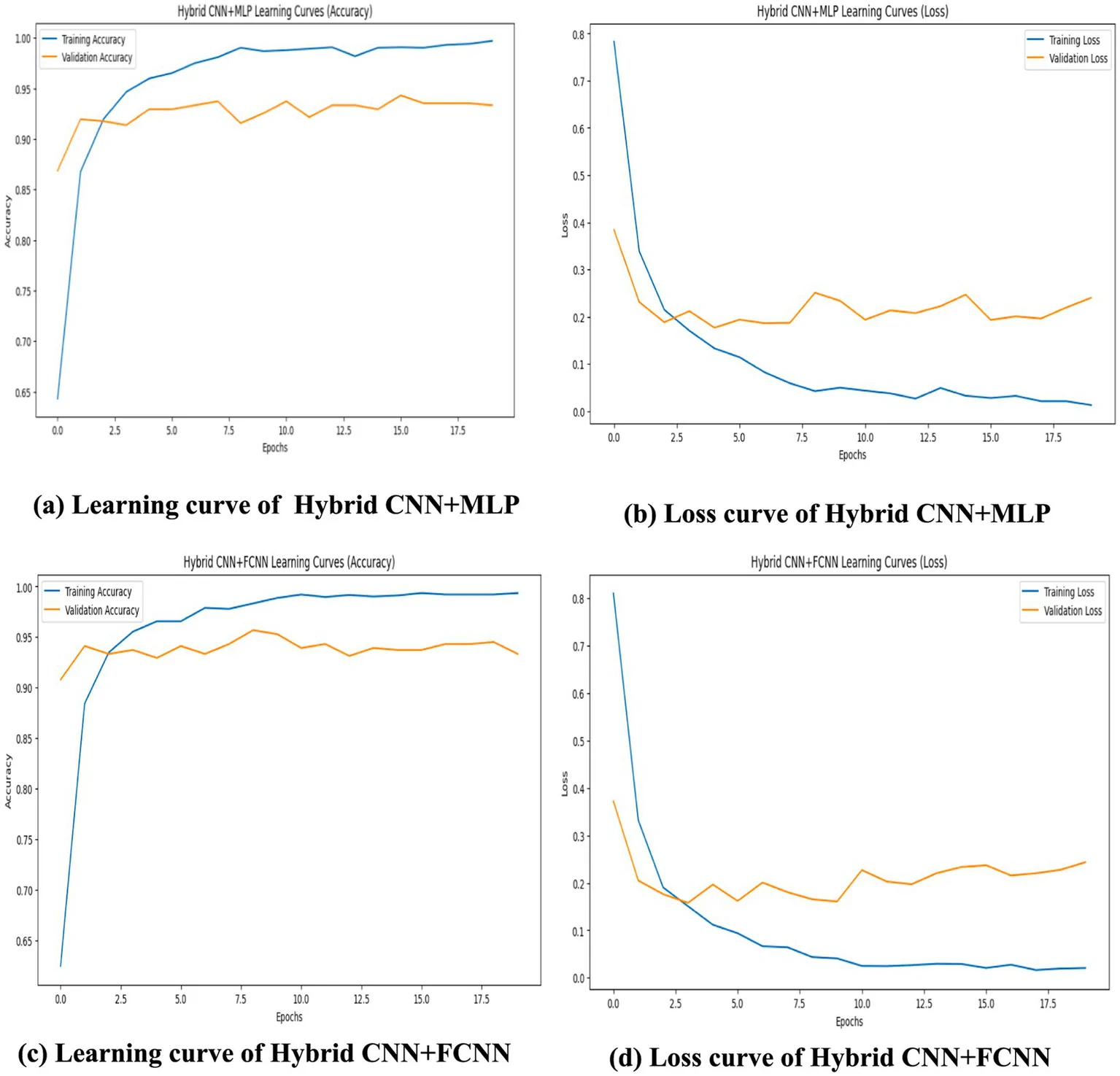
**(A–D)** Learning curves of Hybrid CNN+MLP and Hybrid CNN+FCNN models.

**Figure 11:**
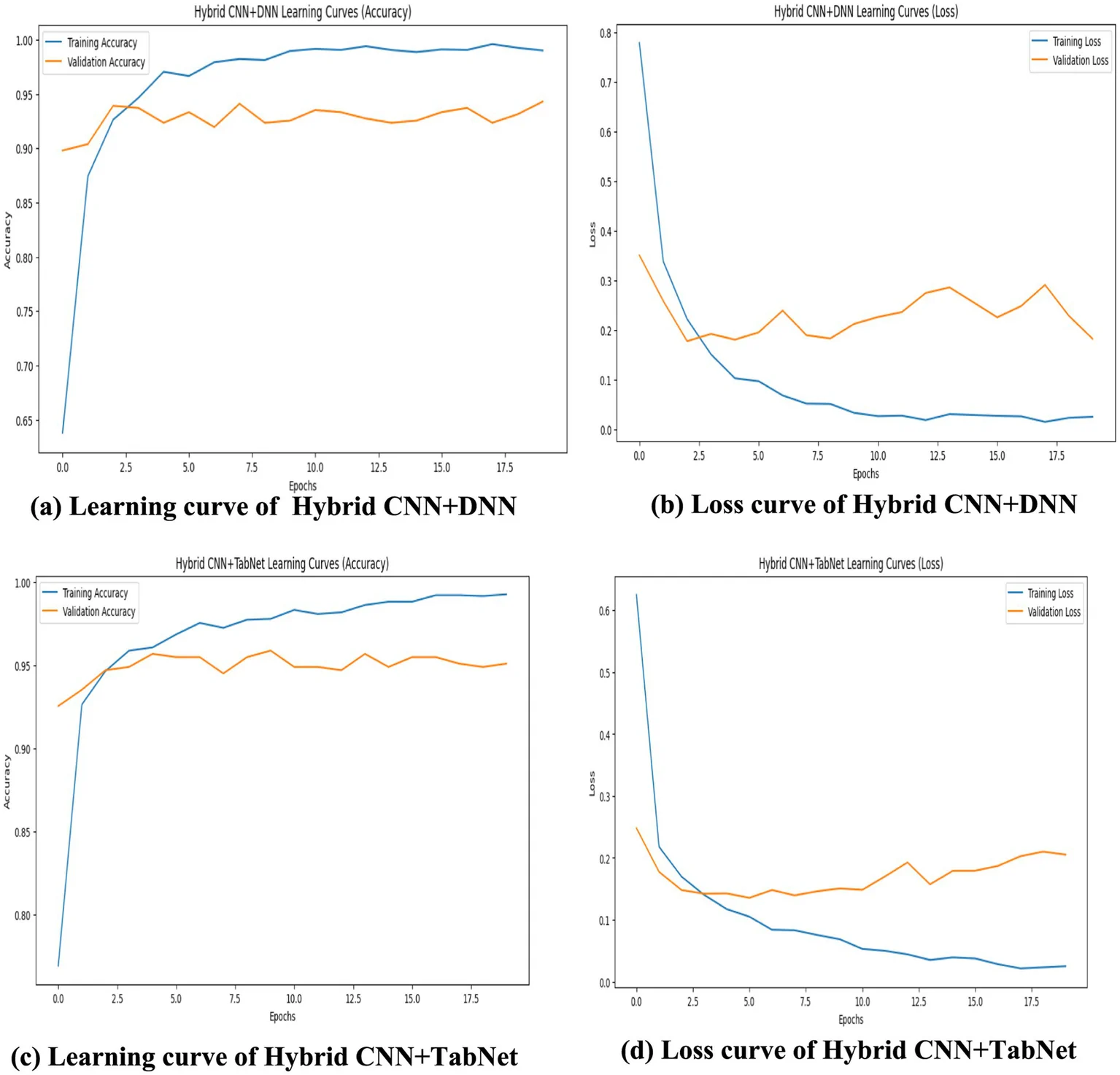
**(A–D)** Learning curves of Hybrid CNN+DNN and Hybrid CNN+TabNet models.

**Figure 12:**
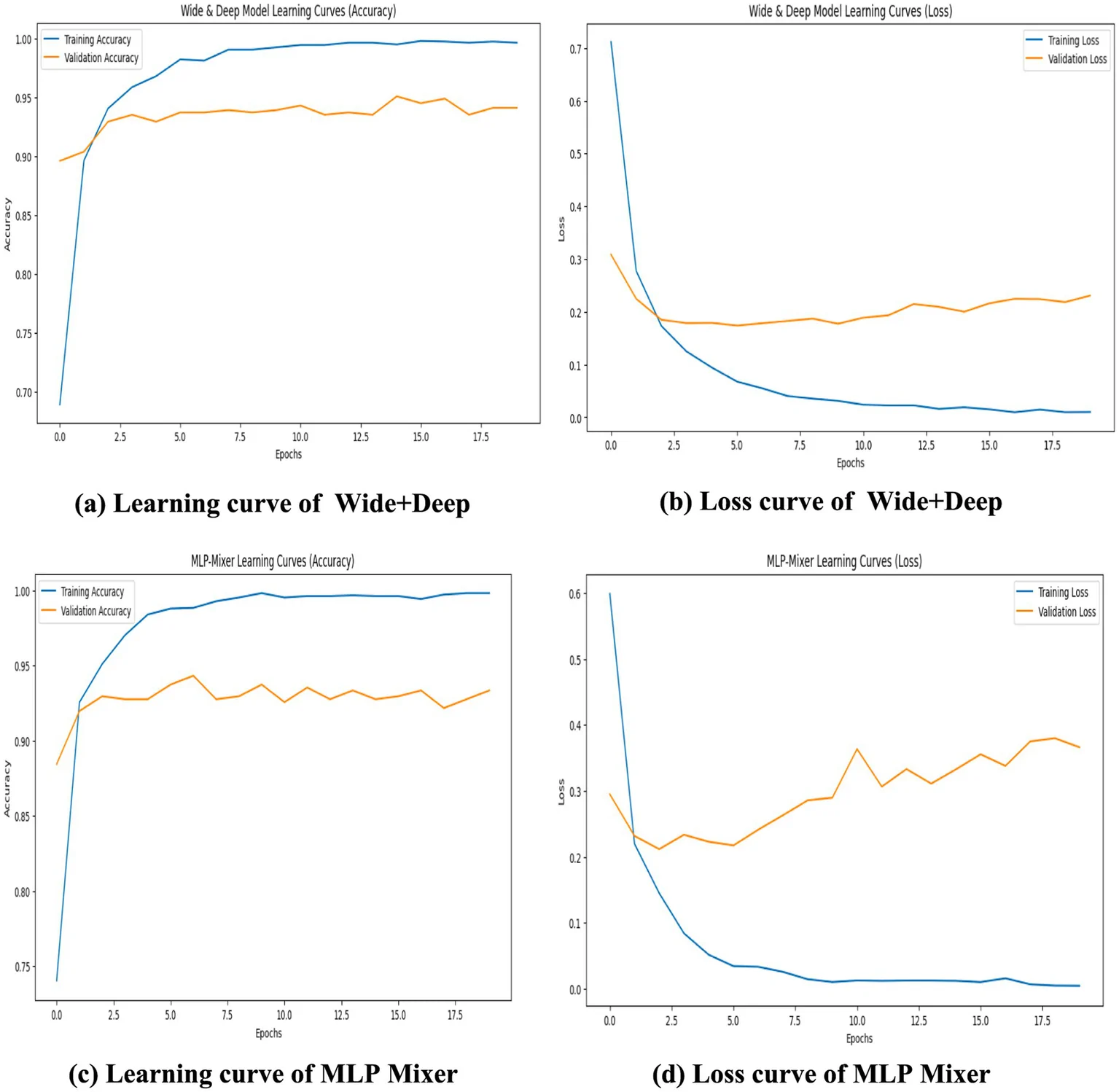
**(A–D)** Learning curves of Wide & Deep and MLP Mixer models.

A set of 15 deep learning and hybrid architectures were tested for classification of the encoded dataset into the three classes found in the dataset: Raw class 1, Raw class 2, and Raw class 3. All training, validation and held-out test measurements presented in this section were calculated with the full 180 feature encoded input representation, unless specified otherwise. The tested architectures were CNN, TabNet, FCNN, Autoencoder, LSTM, Custom CNN, DNN, hybrid CNN based, Wide & Deep, MLP Mixer and Entity Embedding models. Table 7 shows the training accuracy, training loss, validation accuracy, validation loss, held-out test loss and held-out test accuracy for all models evaluated. The precision, recall and F1-scores per class are reported in Table 8. These are not cross validation mean ± SD values, but the values are those used in the single split workflow for archiving purposes. The confusion matrices and AUC–ROC curves are shown for four representative models selected for them; and the complete training and validation learning curves are presented for all 15 models in Figures 5–12. The accuracy and loss of the models on the training set, as well as for the validation and held-out test sets, are presented in Table 7. Table 8 presents the precision, recall and F1-score for each class. Confusion matrices for four representative models are shown in Figure 3, and AUC–ROC curves are shown in Figure 4, and the full training and validation learning curves of all 15 models are shown in Figures 5–12. Table 7 values are not cross-validation mean ± standard-deviation values, but from the archived single-split workflow.

**Table 7:** Training, validation, and held-out test performance of the evaluated models for encoded splice-junction classification.

Sr. no.	Model	Accuracy	Loss	Val_Accuracy	Val_Loss	Test Loss	Test Accuracy
1	CNN	0.988	0.0272	0.949	0.1966	0.1649	0.942
2	TabNet	0.90	0.2347	0.932	0.240	0.287	0.9013
3	FCNN	0.9951	0.0145	0.9314	0.3096	0.3131	0.9451
4	MLP	0.9939	0.0239	0.951	0.2441	0.2494	0.9483
5	Autoencoder	0.9936	0.013	0.9333	0.3677	0.4241	0.9279
6	LSTM	0.5347	0.9869	0.5098	1.0286	1.0372	0.5
7	Custom CNN	0.9838	0.0506	0.9471	0.203	0.1189	0.9687
8	DNN	0.9962	0.0114	0.9412	0.315	0.3208	0.9436
9	Hybrid CNN with MLP	0.997	0.0147	0.9333	0.2401	0.287	0.9216
10	Hybrid CNN with FCNN	0.9925	0.0218	0.9333	0.2437	0.225	0.9373
11	Hybrid CNN with DNN	0.9903	0.023	0.9431	0.182	0.2214	0.931
12	Hybrid CNN with TabNet	0.9929	0.0275	0.951	0.2053	0.2131	0.9436
13	Wide & Deep Model	0.9973	0.0095	0.9412	0.2308	0.2047	0.9498
14	MLP Mixer	0.999	0.0024	0.9333	0.3661	0.3674	0.9373
15	Entity Embedding Model	0.9953	0.0147	0.9392	0.2586	0.251	0.9373

**Table 8:** Class-wise precision, recall, and F1-scores for the three dataset-specific classes.

Sr. no.	Model	Dataset class1			Dataset class2			Dataset class3		
		Precision	Recall	F1-score	Precision	Recall	F1-score	Precision	Recall	F1-score
1	CNN	0.91	0.92	0.91	0.99	0.94	0.96	0.89	0.97	0.93
2	TabNet	0.90	0.89	0.89	0.96	0.88	0.92	0.8	0.96	0.88
3	FCNN	0.93	0.92	0.92	0.97	0.95	0.96	0.92	0.97	0.94
4	MLP	0.92	0.93	0.93	0.98	0.94	0.96	0.91	0.97	0.94
5	Autoencoder	0.91	0.92	0.91	0.97	0.92	0.94	0.87	0.96	0.91
6	LSTM	0.60	0.02	0.03	0.5	0.99	0.67	0.33	0.01	0.01
7	Custom CNN	0.95	0.97	0.96	1	0.96	0.98	0.94	0.99	0.96
8	DNN	0.93	0.91	0.92	0.98	0.95	0.96	0.9	0.97	0.93
9	Hybrid CNN with MLP	0.92	0.88	0.9	0.97	0.91	0.94	0.84	0.98	0.91
10	Hybrid CNN with FCNN	0.92	0.9	0.91	0.99	0.94	0.96	0.87	0.97	0.92
11	Hybrid CNN with DNN	0.94	0.9	0.92	0.96	0.94	0.95	0.88	0.94	0.91
12	Hybrid CNN with TabNet	0.91	0.96	0.93	1	0.91	0.95	0.89	0.99	0.93
13	Wide & Deep model	0.93	0.95	0.94	0.99	0.95	0.97	0.9	0.96	0.93
14	MLP mixer	0.9	0.9	0.9	0.97	0.94	0.95	0.92	0.97	0.95
15	Entity embedding model	0.91	0.93	0.92	0.98	0.93	0.96	0.89	0.95	0.92

All metrics reported in Table 8 are class-wise metrics from the held-out test of the single-split archived. These values do not represent the results of the fold averaging of the cross validation results.

### 5.1 Overall performance analysis

The observed point estimates varied across the evaluated models, as seen in Table 7. Since the archived workflow does not contain a fold-wise cross validation, the differences between models are not considered as statistically tested performance differences but rather described. The highest held-out test accuracy recorded was 96.87% for the Custom CNN model, which was followed by the Wide & Deep model with 94.98% and the MLP with 94.83%. The LSTM model in contrast yielded a held-out test accuracy of 50.00%, which shows that the LSTM configuration used in this experiment is not well suited for the task of classification of encoded splice-junctions. The training accuracy of the MLP Mixer was the highest (99.90%), but the accuracies of the validation and held-out test sets were 93.33 and 93.73%, respectively, as depicted in Table 7. Hence, the high training score was not taken as an indicator of good generalization. Validation behaviour and held-out test performance were used to validate model comparisons, with greater emphasis on the latter than on training accuracy. The workflow did not store any computational profiles. The hardware configuration, wall clock training time, peak memory consumption, energy consumption or inference latency was not measured. Thus, the parameter counts in Table 5 are only model capacity indicators and should not be used for actual feasibility assessment in real time. Specifically the MLP Mixer was the highest in terms of parameter-to-fitting-sample ratio, and its performance on the validation and held-out test sets was worse than the Custom CNN, so the latter is not included as a deployment-ready model.

The best configuration observed among all the tested configurations was the Custom CNN configuration with class-wise F1-scores of 0.972, 0.744, and 0.826, respectively, for classes 0, 1 & 2. with F1-scores of 0.96 (Raw data 1), 0.98 (Raw data 2) and 0.96 (Raw data 3) among the evaluated architectures as depicted in Table 8. For the three classes, the model Wide & Deep obtained F1-scores of 0.94, 0.97 and 0.93, respectively. The MLP Mixer achieved F1-scores of 0.90, 0.95, and 0.95, while the Hybrid CNN + TabNet model achieved F1-scores of 0.93, 0.95, and 0.93. The F1-scores were 0.03 for Raw data1, 0.67 for Raw data 2 and 0.01 for Raw data 3, respectively, for the LSTM as shown in Table 8. This result suggests that the network that was trained with the LSTM was not as effective as the convolutional, dense, and hybrid networks in this encoded sequence-classification problem. The results apply only to the current dataset, pre-processing and evaluation design. They should not be considered as diagnostic, clinical mutation detection, treatment selection, or immediate clinical use at the patient level. The inverse-frequency weights were 1.386395 for raw class 1, 1.386395 for raw class 2, and 0.642092 for raw class 3. All training parameters were kept the same for both conditions (Custom CNN architecture, seed, stratified data partitions, optimizer, batch size, and 20 epochs limit). Values are single corrected stratified split, non-cross validation mean ± std. dev estimates. The inverse-frequency class weighting did not enhance the accuracy, the balanced accuracy or the macro F1 score of the tests in this matched single-split experiment. Test accuracy decreased from 93.73 to 93.26%, balanced accuracy decreased from 94.92 to 94.39%, and macro F1-score decreased from 93.35 to 92.83% as shown in Table 9. However, the class-wise F1-scores decreased marginally in raw classes 1, 2, and 3. Thus, it was not shown that this particular experimental setup provided any advantage in terms of class weighting for the selected Custom CNN.

**Table 9:** Matched unweighted and inverse-frequency class-weighted Custom CNN performance in the corrected stratified single-split experiment.

Class-weighting scheme	Test accuracy	Balanced accuracy	Macro precision	Macro recall	Macro F1	Weighted F1
Unweighted baseline	93.73%	94.92%	92.20%	94.92%	93.35%	93.79%
Inverse-frequency weighted	93.26%	94.39%	91.68%	94.39%	92.83%	93.33%

### 5.2 Class-wise performance analysis

To evaluate the performance of all the architectures, class-wise precision, recall and F1 scores are presented in Table 8. The Custom CNN based on the test set gave the highest observed class-wise F1-scores in the archived single-split evaluation (0.96, 0.98, 0.96 for Raw data 1, Raw data 2 and Raw data 3 test sets, respectively). The LSTM, on the other hand, yielded very inconsistent results by class. It got an F1 score of 0.03 for Raw data 1, 0.67 for Raw data 2 and 0.01 Raw data 3. This pattern is discussed in detail in Section 5.2.1.

#### 5.2.1 LSTM diagnostic analysis

The accuracy of the archived LSTM was low and similar in training, validation, and held-out test sets, suggesting that the LSTM was not able to learn a sufficiently discriminative decision rule for the encoded input representation used in this study. The relatively high loss values and the poor out-of-sample results were consistent with a lack of class separation with the recorded configuration. The results here are not sufficient to conclude that LSTM networks are not appropriate for the analysis of genomic sequences in general. Instead, they showed that the specific LSTM architecture, training setup, and reshaped input representation of 180 features per sample used during the LSTM training in the archived workflow did not yield balanced classification results for the three classes. An alternative LSTM configuration would need to be validated and repeatedly evaluated with leakage control to determine if it performs differently to assess the comparison as shown in Table 10.

**Table 10:** Diagnostic summary of the reported LSTM result.

Diagnostic indicator	Observed result	Interpretation
Input representation	(180, 1)	The complete encoded feature vector was reshaped and treated as a one-dimensional recurrent input.
LSTM configuration	LSTM 128 → LSTM 64 → Dense 128 → Softmax (3)	Archived recurrent baseline configuration.
Training accuracy	53.47%	Limited fitting performance.
Validation accuracy	50.98%	Validation performance remained close to training performance.
Held-out test accuracy	50.00%	Weak three-class discrimination in the archived single-split evaluation.
Training loss	0.9869	Limited reduction in optimization loss.
Validation loss	1.0286	Validation loss remained high.
Held-out test loss	1.0372	Test loss was consistent with weak class discrimination.
Raw class 1 recall/F1-score	0.02/0.03	Raw class 1 was rarely recovered.
Raw class 2 recall/F1-score	0.99/0.67	Predictions were concentrated toward raw class 2.
Raw class 3 recall/F1-score	0.01/0.01	Raw class 3 was rarely recovered.

It should not be concluded that LSTM architectures in general are not applicable to genomic sequence analysis. Instead, it demonstrates that the specific configuration of LSTMs and the encoding of the input for the classification task of this experiment did not acquire a well-balanced decision rule for the current encoded classification problem.

### 5.3 Selected model diagnostic analysis

The four models Custom CNN, Hybrid CNN + TabNet, Wide & Deep, and MLP Mixer are used to illustrate the results of the diagnostic visualizations without repeating the results for every model. These models were chosen to represent the four different types of models, namely convolutional, hybrid, dense-feature, and mixer and to include models with relatively good held-out performance. The confusion matrices for the four models are presented in Figure 3 and the AUC–ROC curves are shown in Figure 4. The full comparative statistics of all 15 architectures are given in Tables 7, 8.

### 5.4 Learning-curve analysis and generalization assessment

The learning curve for training and validation for all 15 evaluated architectures are shown in Figures 5–12. The training and validation accuracy and training and validation categorical cross-entropy loss are plotted against the number of recorded training epochs for each model. These curves offer a direct visual indication of the convergence behaviour and potential for overfitting. The MLP Mixer had a training accuracy of 99.90%, validation accuracy of 93.33%, and held-out test accuracy of 93.73%. The difference between absolute training and test was 6.17 percentage points. The LSTM learning behaviour that was recorded was not the same for the stronger models. The accuracy of the train, validation and train loss was close to 53, 51 and 51%, respectively. These trajectories reflect a model that learned few discriminative class boundaries instead of a model that first performed well on the training data and then overfitted. So the LSTM result reported was assumed to be a failure mode specific to the configuration, not a failure mode of the recurrent neural network in general.

This suggests that the model learned the data training set better than it did the data that was not used in training. As a result, the accuracy of training was not considered for model ranking. In all architectures, if training loss continued to decrease without a corresponding decrease in validation loss, then it was interpreted as an increased risk of overfitting. The ultimate performance of the models was thus highlighted in terms of validation-loss behaviour, held-out test performance and class-wise F1-scores.

### 5.5 Performance of traditional machine learning baselines

To assess the performance of traditional machine learning classifiers to classify the encoded benchmark set of 3,186 instances of splice-junction sequences, further experiments were carried out. As per Table 11, the classifiers that were evaluated - Logistic Regression, Support Vector Machine (SVM), K-Nearest Neighbors (KNN), Decision Tree, Random Forest, Extra Trees, AdaBoost, and Gradient Boosting. To ensure uniform experimental conditions, a 180-feature encoded representation was used for training and evaluation of all classifiers and a fixed train-test split was used. The accuracy, the weighted precision, the weighted recall and the weighted F1-score were used to evaluate the model performance. The class distributions in the benchmark dataset are not equal, which meant that weighted-average metrics were chosen to weight each class by the number of instances.

**Table 11:** Performance of traditional machine learning classifiers.

Model	Accuracy (%)	Precision (%)	Recall (%)	F1-score (%)
Logistic Regression	93.26	93.4	93.26	93.29
Support Vector Machine (SVM)	95.92	96.01	95.92	95.94
Random Forest	96.39	96.45	96.39	96.41
Gradient Boosting	95.92	95.98	95.92	95.94
K-Nearest Neighbors	74.29	78.83	74.29	74.15
Decision Tree	90.91	90.94	90.91	90.92
AdaBoost	93.89	94.16	93.89	93.93
Extra Trees	96.08	96.12	96.08	96.09

The experimental results show that the majority of the machine learning classifiers evaluated were effective on the encoded splice-junction benchmark dataset. The best accuracy (96.39%), Weighted precision (96.45%), Weighted recall (96.39%), and Weighted F1-score (96.41%) were obtained by the Random Forest model. The Extra Trees also yielded good results, accuracy of 96.08% and an F1 score of 96.09%. The SVM model and the Gradient Boosting model both had an accuracy of 95.92% and F1 score of 95.94%. Logistic Regression and AdaBoost also showed good classification results, with accuracies greater than 93%. A single model (Decision Tree) was less effective than the ensemble-based models (Tree classifiers) and achieved an accuracy of 90.91%. The worst performance was by KNN, which had an accuracy of 74.29% and an F1-score of 74.15%. The result indicates that distance-based classification might not be appropriate in this dataset for the encoded feature representation used, being of high dimension. This performance degradation can be related to the fact that KNN is sensitive to the dimensionality of the features, to the distribution of local data and to the choice of the neighbouring instances. The results show that ensemble based classifiers, in particular, Random Forest and Extra Trees are suitable for the encoded splice-junction classification task. SVM and Gradient Boosting also provided consistently high performance. The results demonstrate the effectiveness of classic machine learning algorithms applied to the current best-in-class dataset and serves as a reference for future experiments using encoded splice-junction sequences. These results could be more robustly and generalisably determined with further evaluation and use of larger independent datasets, repeated cross validation, and statistical significance testing.

## 6 Comparison with prior work, limitations, and future work

### 6.1 Interpretation of comparative results

The observed performance values on the encoded splice-junction dataset were different for the evaluated architectures. During the single split evaluation in the archive, the Custom CNN resulted in the largest held-out test accuracy seen, with the Wide & Deep and MLP models following. Such comparisons are descriptive, and do not constitute statistically significant evidence of superiority of one model over other models and/or over previously published studies as depicted in Table 12.

**Table 12:** Descriptive comparison of reported accuracies in previous research and held-out test in the current evaluation.

Ref. no.	Technique used	Dataset used	Accuracy (%)	Evaluation basis	Clinical features considered	outcome
Gautam et al. (2025)	CNN	splice-junction gene dataset	95	Reported by cited study	No	Sequence pattern recognition; no clinical linkage reported
Magnini et al. (2022)	AI-based gene detection	splice-junction gene dataset	93.4	Reported by cited study	No	General sequence detection; no clinical interpretation reported
Loka et al. (2019)	2D CNN on 1D encoding	DNA sequence dataset	96.5	Reported by cited study	No	Pattern extraction study
Anusuya and Gomathi (2021)	Hybrid ML	Splice-junction gene dataset	93.7	Reported by cited study	No	Sequence-based classification
Maigari et al. (2025)	GAN	Splice-junction dataset	96.8	Reported by cited study	No	Sequence-generation/classification study
Azmi and Berrado (2021)	Lasso-based pruning	DNA sequence dataset	91.2	Reported by cited study	No	Feature-reduction study
Marchand et al. (2023)	Sample re-attribution mitigation	DNA classification	92.3	Reported by cited study	No	Data-balancing study
Konovalenko (2019)	Heuristic-based ML algorithm	Splice-junction dataset	94.7	Reported by cited study	No	Model-efficiency study
Present study	Custom CNN	Encoded splice-junction dataset	96.87	Held-out test accuracy	No	Highest observed held-out test accuracy in the current evaluation
Present study	Wide & Deep	Encoded splice-junction dataset	94.98	Held-out test accuracy	No	Second-highest observed held-out test accuracy in the current evaluation
Present study	MLP	Encoded splice-junction dataset	94.83	Held-out test accuracy	No	Third-highest observed held-out test accuracy in the current evaluation

Since a single archived evaluation split was used and not all per-sample prediction records across all final models were saved, the retrospective formal paired statistical comparisons were not performed. The results reported are thus from the current dataset, representation of features, preprocessing pipeline, model architecture, and evaluation log. The models need to be tested with repeated stratified cross validation, only training the preprocessing, random seeds, saving predictions out of the fold, and using the right type of pairwise statistical test for future work. In the additional comparison with the conventional machine learning baseline, the Random Forest, Extra Trees, SVM and Gradient Boosting models were found to be competitive on the relatively small encoded dataset. The highest conventional baseline with 96.39% was achieved by Random Forest while the Custom CNN achieved an accuracy of 96.87%. The reported single-split evaluation thus indicates a difference of 0.48 percentage points for the two models, but this should be considered descriptive and not as a statistically proven superiority of the Custom CNN. The results show that conventional ensemble and kernel-based classifiers can achieve comparable performance with much richer deep learning architectures on the state-of-the-art dataset. The main objective of the study is a computational benchmarking of several model families under a common set of experiments, and not a significant performance gain or some architectural innovation.

Different performance values were observed across the evaluated models from the current encoded splice-junction dataset. These results are only descriptive and should not be considered as statistically significant superiority, clinical ability, or clinical diagnostic value.

### 6.2 Limitations and future work

There are a number of limitations to this study. In the present results, the classification is limited to a public encoded benchmark set, which can be computed. They are not intended as a measure of clinical diagnostic performance or disease-specific mutation detection, treatment selection, clinical utility or readiness for deployment. The findings of the present study are restricted to an encoded benchmark dataset of splice junctions, and should not be used as evidence of clinical diagnostic performance. Substantial additional validation and governance would be necessary before any real-world use of a translation of sequence-classification models into clinical genomics. First, future studies would require patient-level validation of the same samples of the genome collected independently, with relevant clinical and demographic data provided. The data gathered for such evaluation should be from a variety of populations, sequencing platforms, and disease settings to evaluate for robustness and generalizability. Second, the interpretability of the models should be explored through a proper explanation methodology, including feature-attribution methods and attention-based analysis, to assess the ability to meaningfully analyze model decisions from the domain experts’ point of view. A systematic feature selection, feature attribution and feature ablation was not performed in the present study. All available encoded features were kept in the reported workflow, but the contribution of individual columns to the results was not quantified towards the information gain, SHAP value, permutation importance, etc. Future work should conduct leakage-controlled experiments to compare the full set of 180 features with pre-defined and data-driven reduced sets of features. Only feature selection should be done on training data; and, only tested on independent validation and held-out test data.

Another restriction is that the archived workflow used for the 15-model comparison standardized the features prior to the creation of the held-out test partition. This sequence might have been responsible for introducing information leakage as the distribution in the test-set was a factor in the parameters of the scaling. The experiment to target the class weights was implemented using a corrected split-first, training-only scaling procedure, but not all the archived model configurations were run with the correct scaling. In future comparisons, all transformations should be fit only on the training data and leakage controlled preprocessing should be used with all models. This preprocessing sequence may lead to leakage of information since the test distribution is used in the process of scaling. Future experiments should involve partitioning of data, fitting all of the preprocessing transformations only to the training partition, then applying them without modification to the validation and held-out test partitions. Not all the results of the five-fold cross-validation were recorded in the archived evaluation record. We therefore report single-split training and validation and held-out test values instead of mean ± standard deviation values from cross-validation. All models should be tested in future with stratified repeated cross validation or nested cross validation, where the preprocessing, feature selection and hyperparameter optimization are done only within training folds. This would give a more solid foundation to estimate the variability, to compare the robustness of the models, and to prevent accidental exposure to the test-set.

The other comparisons of conventional machine learning baselines demonstrated that Random Forest, Extra Trees, SVM and Gradient Boosting continued to be competitive on the relatively small encoded dataset. The best performing baseline method was Random Forest, with an accuracy of 96.39% and the Custom CNN achieved 96.87% accuracy. Therefore, the reported single-split evaluation result difference of 0.48 percentage points is not a statistically determined superiority of the Custom CNN, but merely a description of the difference observed during the evaluation. The results show that traditional classifiers (ensemble and kernel) can achieve comparable performance to significantly more complex deep learning architectures on the current benchmark dataset. The key result of the study, therefore, is not the high performance of any given model family or the new architecture, but the fact that several model families can be compared in the same experimental context. The novelty of the study, therefore, is not a significant performance gain, but the ability to compare multiple model families in a common experimental setting.

## 7 Conclusion

The study showed a comparison of 15 deep learning and hybrid architectures for classification of an encoded public splice junction dataset. For the single-split evaluation, the Custom CNN achieved the highest held-out test accuracy of 96.87%, and class-wise F1-scores of 0.96, 0.98, and 0.96 for the three classes in the dataset. The results shown are descriptive and are based on the data and evaluation methodology used in the current database. They do not show significant differences between models, differences over previous studies, clinical diagnostic value or clinical application. Before making more general statements, independent data, leakage controlled repeated validation, complete prediction records, statistical comparison of model outputs and clinically validated outcome data would be needed.

## Data Availability

Publicly available datasets were analyzed in this study. This data can be found at: https://archive.ics.uci.edu/dataset/69/molecular+biology+splice+junction+gene+sequences.
